# A Study of the Effects of Al, Cr, Hf, and Ti Additions on the Microstructure and Oxidation of Nb-24Ti-18Si Silicide Based Alloys

**DOI:** 10.3390/ma11091579

**Published:** 2018-09-01

**Authors:** Jack Nelson, Mohammad Ghadyani, Claire Utton, Panos Tsakiropoulos

**Affiliations:** Department of Materials Science and Engineering, Sir Robert Hadfield Building, The University of Sheffield, Mappin Street, Sheffield S1 3JD, UK; pha05jn@gmail.com (J.N.); m.ghadyani@sheffield.ac.uk (M.G.); c.utton@sheffield.ac.uk (C.U.)

**Keywords:** Niobium alloys, intermetallics, solid solution, oxidation, hardness, Young’s modulus

## Abstract

In Nb-silicide based alloys Al, Cr, Hf, and Ti additions are crucial for achieving balance of properties. It is not known how the simultaneous addition of Hf with Al and Ti, or Hf with Al, Cr, and Ti affects macrosegregation, and how the alloying affects hardness, Young’s modulus and bulk alloy oxidation, and contamination of the solid solution Nb_ss_ and the Nb_5_Si_3_ compound by oxygen. Two alloys with nominal compositions (at.%) Nb-24Ti-18Si-5Al-5Hf (alloy NbSiTiHf-5Al) and Nb-24Ti-18Si-5Al-5Cr-5Hf (alloy NbSiTiHf-5Al-5Cr) were studied in the as-cast and heat-treated conditions and after isothermal oxidation at 800 and 1200 °C and were compared with similar alloys without Hf. In both alloys there was macrosegregation of Si and Ti, which was more severe in NbSiTiHf-5Al. Both alloys formed Nb_ss_+βNb_5_Si_3_ eutectic. The Nb_ss_ was stable and its Al and Cr concentrations increased with increasing Ti concentration. In both conditions the βNb_5_Si_3_ was observed in the alloys NbSiTiHf-5Al and NbSiTiHf-5Al-5Cr, and the γNb_5_Si_3_ only in the alloy NbSiTiHf-5Al. In both heat-treated alloys, separate Hf-rich Nb_5_Si_3_ grains were formed. The Si and Al concentrations in Nb_5_Si_3_ respectively decreased and increased with increasing Ti concentration. Al and Cr had a stronger hardening effect in the Nb_ss_ than Al, Cr, and Hf. Al, Cr, and Ti had a stronger negative effect on the Young’s modulus of the Nb_ss_ compared with Al, Cr, Hf, and Ti. When Nb was substituted by Ti, Cr, and Hf, and Si by Al in the βNb_5_Si_3_, the Young’s modulus was reduced compared with the unalloyed silicide. At 800 °C both alloys did not exhibit catastrophic pest-oxidation after 100 h. The Nb_ss_ and Nb_5_Si_3_ were contaminated by oxygen in both alloys, the former more severely. At 1200 °C the scales spalled-off, more severely in the alloy NbSiTiHf-5Al, where substrate that was heavily contaminated by oxygen below the scale also spalled-off. In both alloys the contamination of Nb_5_Si_3_ and Nb_ss_ by oxygen was more severe compared with 800 °C, but the silicides were not contaminated by oxygen in their bulk. The Nb_ss_ was not contaminated by oxygen only in the bulk of the alloy NbSiTiHf-5Al-5Cr.

## 1. Introduction

Niobium silicide based alloys can offer a balance of low, intermediate, and high temperature properties and are candidate materials to replace Ni-based superalloys in future aero-engines, to enable the latter to meet environmental and performance targets [1]. The latter have been set by industry and are property goals for new ultra-high temperature alloys with capabilities beyond those of Ni-based superalloys: the room temperature fracture toughness must be above 20 MPa(m)^1/2^, there must be less than 1% creep in 125 h at 1200 °C and σ > 170 MPa (alloy density ρ = 7 g/cm^3^), and the oxidation life at 1315 °C must be equal to that of second generation single crystal Ni-based superalloys at 1150 °C with a short term oxidation goal to have sufficient oxidation resistance in the uncoated condition to survive under typical engine conditions, which requires a loss of material less than 200 µm thickness in 10 h at 1370 °C and a long term oxidation goal that requires a loss of material less than 25 µm thickness in 100 h at 1315 °C [1]. Rotor weight savings relative to Ni superalloy baseline are estimated to be about 20%, 22%, and 21% for Nb-silicide based blades, respectively, in advanced high pressure turbine, current high pressure turbine, and current low pressure turbine [1]. 

The most important phases in the microstructures of these new alloys are considered to be the bcc Nb solid solution (Nb_ss_) and the tetragonal Nb_5_Si_3_ compound. The latter can form as the high temperature βNb_5_Si_3_ (*tI*32, W_5_Si_3_-type, D8_m_) or the low temperature αNb_5_Si_3_ (*tI*32, Cr_5_B_3_-type, D8*_1_*) silicide and is important for the strength and creep of the alloys. The Nb_ss_ is important for the fracture toughness of the alloys and plays a key role in their oxidation and creep properties. A high volume fraction of the Nb_ss_ has a negative effect on oxidation resistance, high temperature strength, and creep. A high volume fraction of Nb_5_Si_3_ is desirable for creep resistance but can be detrimental to fracture toughness. Bewlay et al. [1] reviewed the progress on Nb-silicide alloy development till 2002. More recently, one of the authors of this paper reviewed the alloying behaviour and properties of the key phases in Nb-silicide based alloys and showed how such data can be used for the design and/or selection of new alloys [2]. It was also shown that some of the alloy compositions, some of the bcc Nb solid solution compositions, and some of the Nb_ss_+Nb_5_Si_3_ eutectic compositions satisfy the standard definition of the so called High Entropy Alloys (HEA) [2]. 

Resistance to oxidation is essential for materials envisioned for high temperature applications in aero-engines. Since the early stages of the development of Nb-silicide based alloys, it was recognized that a research priority should be the improvement of their oxidation. The latter has been achieved with alloy development strategies that used additions of Al, Cr, Hf, and Ti individually or simultaneously in Nb-silicide based alloys [2,3,4,5]. Improvements of oxidation behaviour have been sought with additions of B, Ce, Ge, and Sn [3,4,5,6,7]. As has been the case with Ni-based superalloys, the Nb-silicide based alloys also will require coatings. Coating development has considered Cr and Y modified silicide coatings (for example see [8,9,10]), Mo-Si-Al coatings (for example see [11,12]), and multilayer oxidation resistant coatings (for example see [13,14]). To date most of the research has been on arc melted alloys that have been studied in their cast and heat-treated conditions. There is very little research on directionally solidified alloys and most of it is on alloys processed using optical floating zone (OFZ) melting. There is even less research on powder metallurgy alloys and on alloys produced using spark plasma sintering [2,15,16,17,18]. 

When added individually to Nb, Al and Cr have a strong effect on its ductile to brittle transition temperature (DBTT), which is increased significantly above −50 °C, compared with Hf and Ti that have a weak effect [19]. Aluminium and Cr were added to Nb-silicide based alloys owing to the properties of their oxides, and Ti because it provides solid solution strengthening to the Nb_ss_, significantly improves oxidation [1,2,5,20] and fracture toughness [21], and reduces density [5]. The maximum solid solubility of oxygen in Nb is about 9 at.% at 1915 °C [22]. Hafnium was added to scavenge dissolved oxygen in the alloys [4,5]. High concentrations of Ti and/or Hf in the alloy and Nb_5_Si_3_ could stabilise the hexagonal γNb_5_Si_3_ (*hP*16 Mn_5_Si_3_-type D8_8_) in the microstructure of Nb-silicide based alloys, which is undesirable owing to its inferior creep compared with the tetragonal Nb_5_Si_3_. When the Nb/(Ti+Hf) ratio in the alloy and Nb_5_Si_3_ is less than one, the likelihood of the γNb_5_Si_3_ being stable increases significantly. Higher values of this ratio are desirable for creep resistance [2,23]. The concentrations of Al, Cr, Hf, and Ti in Nb-silicide based alloys must be optimised in order (i) to achieve a balance of mechanical properties and oxidation, in particular fracture toughness, high temperature strength, and creep and (ii) to suppress pest oxidation. 

To date, most of the studied Nb silicide based alloys with Al, Cr, Hf, and Ti additions also had additions of other transition metals, refractory metals, simple metals, and metalloid elements (examples of alloy compositions are given in Supplemental data), which makes it very difficult to clarify how Hf affects microstructure, oxidation, and other properties. Bewlay et al. studied different directionally solidified Nb-Ti-Hf-Si alloys with 21 < Ti < 33 at.%, 7.5 < Hf < 12.5 at.%, and 0.85 < Nb/(Ti+Hf) < 1.95 and reported a low temperature eutectoid phase transformation of Nb_3_Si to Nb_ss_ and hexagonal hP16 Nb_5_Si_3_ instead of the Nb_ss_ and tetragonal tI32 Nb_5_Si_3_ that is observed in binary Nb-Si alloys [24]. The effects on microstructure and properties of the simultaneous addition of (i) Al and Ti; (ii) Cr and Ti; (iii) Al, Cr and Ti; and (iv) Hf and Ti in Nb-18Si silicide based alloys (all compositions in this paper are given in at.% unless stated otherwise) without other transition and simple metals, and refractory and metalloid element additions were reported in [5,25,26]. For example, it was shown that Al significantly improved the oxidation of the Nb-24Ti-18Si-5Al alloy at 800 °C compared with the alloy Nb-24Ti-18Si-5Cr and suppressed the Nb_3_Si compound and the Nb_ss_ +Nb_3_Si eutectic that was replaced by the Nb_ss_+βNb_5_Si_3_ eutectic [25,27]. However, research has not provided answers to the following questions. (1) How the synergy of Hf with Al and Ti, or with Al, Cr and Ti would affect the microstructure and properties of Nb-silicide based alloys? (2) What would be the effect of these synergies on the microstructure, such as macrosegregation of Si, the type of eutectic formed, the prototype of the primary Nb_5_Si_3_, and the alloying behaviour of the Nb_ss_ and Nb_5_Si_3_ phases? (3) What would be the effect of these synergies on the oxidation properties of the alloys, or on physical properties such as hardness and Young’s modulus? The motivation for the research presented in this paper was to address these three questions by focusing on alloys based on Nb-24Ti-18Si. The answers to the above questions are essential for the design of new more advanced alloys for high temperature applications [2]. 

The structure of the paper is as follows. In order to provide answers to each of the above three questions, first new experimental results are presented for two alloys with nominal compositions Nb-24Ti-18Si-5Al-5Hf (alloy NbSiTiHf-5Al) and Nb-24Ti-18Si-5Al-5Cr-5Hf (alloy NbSiTiHf-5Al-5Cr). Then the microstructures and oxidation of these alloys are compared with alloys studied previously. Finally, the effects of alloying additions on the macrosegregation of Si, the chemical composition of the Nb_5_Si_3_ and Nb_ss_ and their properties, and the oxidation of the alloys and contamination of phases by oxygen are discussed. 

## 2. Experimental

Small buttons (20 g) of the alloys NbSiTiHf-5Al and NbSiTiHf-5Al-5Cr were prepared using high purity elements and arc melting with a non-consumable tungsten electrode and a water cooled copper crucible in an inert atmosphere and were heat treated in an Ar atmosphere for 100 h, as described in [25]. The concentrations of oxygen, carbon, and nitrogen in these alloys were not determined. In arc melted Nb-silicide based alloys studied in our group, typical values of the concentrations of the above elements are in the ranges 0.14 to 0.19 at.% O, 0.04 to 0.08 at.% C, and 0.04 to 0.08 at.% N. Specimens for heat treatment were cut from the bulk of the buttons. The cast and heat treated (1300 °C/100 h for the alloy NbSiTiHf-5Al and 1500 °C/100 h for the alloy NbSiTiHf-5Al-5Cr) microstructures were characterised using powder or bulk X-ray diffraction (XRD, Siemens D5000, Hiltonbrooks Ltd, Crew, UK), scanning electron microscopy (SEM, FEI Inspect F50, ThermoFisher Scientific, Hillsboro, OR, USA), SEM with energy dispersive analysis (EDS) with well-polished elemental standards (JEOL 6400, JEOL Ltd., Tokyo, Japan and Philips XL30S, ThermoFisher Scientific, Hillsboro, OR, USA) and electron probe micro-analysis (EPMA, JEOL JXA8230, JEOL Ltd., Tokyo, Japan) with wavelength-dispersive spectroscopy (WDS). The EPMA analyses were carried out as described in [5]. Our group uses EPMA to determine whether the microstructures in the bulk of cast Nb-silicide based alloys are contaminated by oxygen. The phases were determined using WINX^POW^ software (STOE, Darmstadt, Germany) and ICDD PDF-4+2012 database packages software (ICDD, Newtown Square, PA, USA). The isothermal oxidation of the alloys in air at 800 °C and 1200 °C was studied using cubic specimens (3 × 3 × 3 mm^3^) in a NETZSCH STA 49 F3 thermal analyser (NETZSCH GmbH, Selb, Germany). The specimens were cut from the bulk of the cast buttons.

The microstructures of the oxidised alloys in the bulk and below the scale were also studied in order to assess the contamination of the microstructure by interstitials and whether any of the phases present were immune to this contamination. For the alloy NbSiTiHf-5Al-5Cr, the nano-hardness and Young’s modulus of the Nb solid solution (Nb_ss_) and Nb_5_Si_3_ were measured as described in [28] using a Berkovitch indenter in a Hysitron Triboscope TS70 nano-mechanical test instrument (Hysitron, Minneapolis, MN, USA) attached to a Veeco dimension 3100 atomic force microscope (Veeco Instruments Inc, Santa Barbara, CA, USA). Serpentine runs, typically 8 × 8, were run in areas of the bulk microstructure using a 4000 µN load. The microhardness of the phases was measured using a Mitutoyo hardness machine (Mitutoyo America, Aurora, IL, USA) with a load of 0.05 kg.

## 3. Results

### 3.1. Cast Alloys

The actual compositions of the two alloys were Nb-22.3Ti-21.3Si-4.5Al-5.4Hf (alloy NbSiTiHf-5Al) and Nb-23.5Ti-20.1Si-5.5Al-4.5Cr-5.2Hf (alloy NbSiTiHf-5Al-5Cr). Both alloys were richer in Si compared with their nominal compositions. The cast alloys exhibited macrosegregation of Si and Ti, the values of which were 7.4 at.% Si and 5 at.% Ti for the alloy NbSiTiHf-5Al, and 4 at.% Si and 1.9 at.% Ti for the alloy NbSiTiHf-5Al-5Cr, where the macrosegregation of element X is given as the difference between its maximum and minimum measured concentration (MACX = C_max_ – C_min_) [29]. 

The cast microstructures of both alloys consisted of the Nb_ss_ and Nb_5_Si_3_ phases and Nb_ss_+βNb_5_Si_3_ eutectic. In both alloys the eutectic was fine and was formed in the top and in the bulk of the buttons. In the bulk the volume fraction of the eutectic was higher. In both alloys no eutectic was observed in the areas of the button where the melt had solidified under the highest cooling rates. There was Hf rich Nb_5_Si_3_ in both alloys and Hf rich Nb_ss_ in the alloy NbSiTiHf-5Al-5Cr. In the alloy NbSiTiHf-5Al the Hf rich Nb_5_Si_3_ was observed in the very bottom of the button where the melt was in direct contact with the copper crucible and the “normal” Nb_5_Si_3_ was observed in the transition from bottom to bulk and in the bulk and top of the ingot. The Hf rich Nb_5_Si_3_ exhibited brighter contrast under back-scatter electron imaging (BSE) compared with the “normal” Nb_5_Si_3_ owing to its higher concentration of Hf. The partitioning of Hf in the microstructures of both alloys made their characterisation using BSE imaging challenging; phases could not be distinguished on the basis of their contrast, in particular the Nb_ss_. 

**Figure 1:**
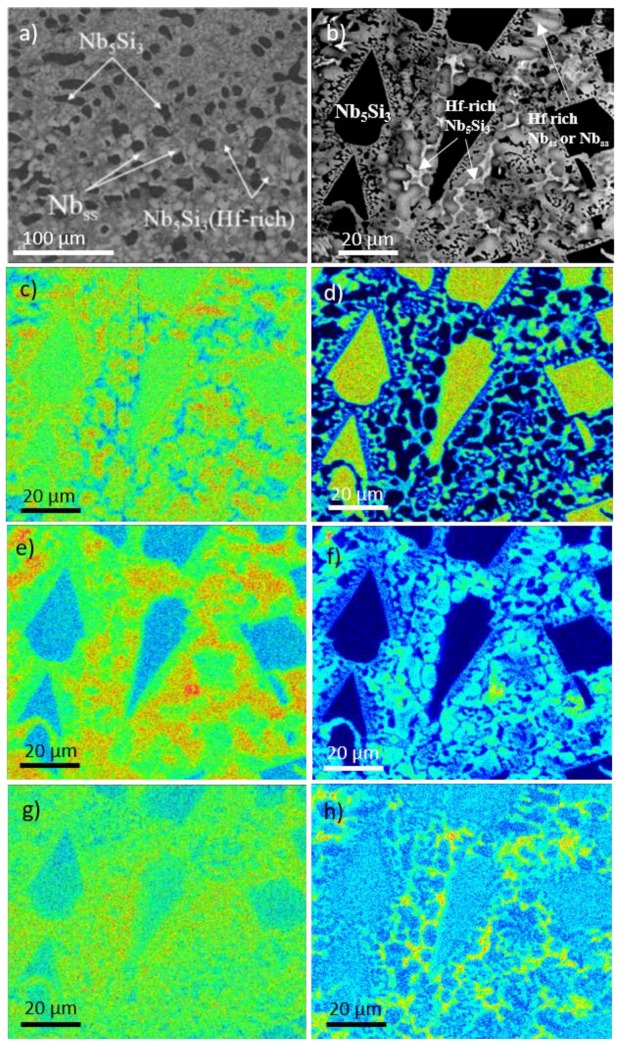
BSE images of the bulk of the cast alloys NbSiTiHf-5Al (**a**) and NbSiTiHf-5Al-5Cr (**b**) and EPMA X-ray maps corresponding to (**b**) where (**c**) Nb, (**d**) Si, (**e**) Ti, (**f**) Cr, (**g**) Al and (**h**) Hf.

The chemical analysis data are given in Table 1 and Table 2 for the alloys NbSiTiHf-5Al and NbSiTiHf-5Al-5Cr, respectively. The data reported in these Tables were taken from all regions of the cast buttons. Table 1 and Table 2 (and Tables S1 and S2 in Supplemental data) give the average concentration, standard deviation, and the minimum and maximum analysis values. The typical microstructure in the bulk of the alloy NbSiTiHf-5Al is shown in Figure 1a. Figure 1b shows the microstructure in the bulk of the alloy NbSiTiHf-5Al-5Cr and Figure 1c to h show EPMA elemental maps. The powder XRD data confirmed that both βNb_5_Si_3_ (*tI*32 W_5_Si_3_-type D8_m_) and γNb_5_Si_3_ (*hP*16 Mn_5_Si_3_-type D8_8_) were present in the microstructure of the alloy NbSiTiHf-5Al and only the βNb_5_Si_3_ in the alloy JN1 (Figure S1 in Supplemental data). There was no evidence of the C14-NbCr_2_ Laves phase in the Cr containing alloy NbSiTiHf-5Al-5Cr.

The Hf rich Nb_5_Si_3_ and Nb_ss_ can be distinguished in the elemental maps in Figure 1c–h. In these maps the red areas have a higher concentration of the analysed element, and in the blue areas there is little or none of the analysed element. The Si and Nb maps clearly show the Nb_5_Si_3_ grains. The Hf rich regions align with the low Nb and the high Si regions, which indicate the Hf rich Nb_5_Si_3_. The Hf rich Nb_5_Si_3_ regions were found in between the Nb_5_Si_3_ grains and the Nb_ss_. The Ti and Cr rich regions were also rich in Hf. The Al map shows that the concentration of this element was higher in the Nb_ss_ compared with the Nb_5_Si_3_. The Cr map does not provide convincing evidence for the presence of the C14-NbCr_2_ Laves phase, with the exception of one area which was also rich in Ti. 

The Al and Hf concentrations in the Nb_ss_ were essentially the same in the two alloys, and the concentrations of Cr and Ti increased in the Hf-rich Nb_ss_ in the alloy NbSiTiHf-5Al-5Cr. The concentrations of Al in the Nb_5_Si_3_ and Hf rich Nb_5_Si_3_ were the same in both alloys but those of Hf and Ti were higher in the alloy NbSiTiHf-5Al-5Cr. The Al+Si concentration of the eutectic was 20.6 and 18.9 at.% in the alloys NbSiTiHf-5Al and NbSiTiHf-5Al-5Cr, respectively. 

### 3.2. Heat Treated Alloys

After the heat treatments the average compositions of the heat treated specimens were Nb-21.3Ti-20.5Si-4.3Al-5.7Hf and Nb-24.1Ti-19.3Si-3.8Al-4.1Cr-5.7Hf, respectively, for the alloys NbSiTiHf-5Al and NbSiTiHf-5Al-5Cr (see Table 1 and Table 2). In both alloys chemical inhomogeneity of Si and Ti was still present and the concentrations of these elements were in the ranges 25.4 to 16.3 at.% Si and 25.1 to 19.2 at.% Ti in the alloy NbSiTiHf-5Al, and 21.2 to 15.3 at.% Si and 25.1 to 22.9 at.% Ti in the alloy NbSiTiHf-5Al-5Cr.

**Figure 2:**
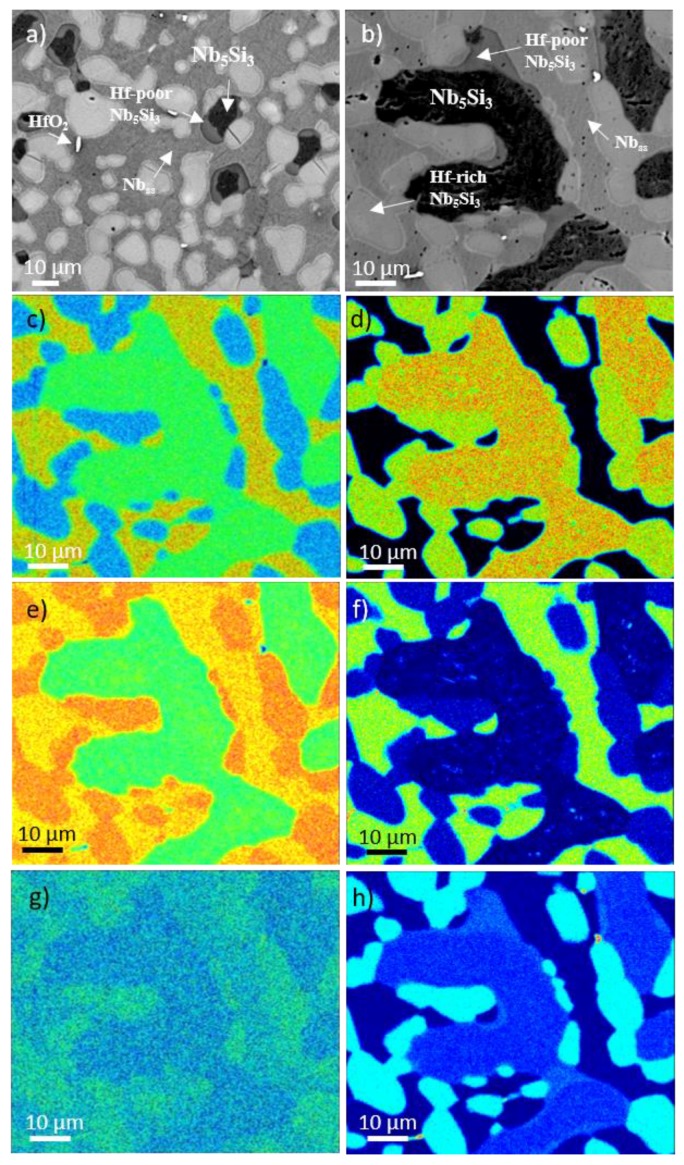
BSE images (**a**) and (**b**) of the heat treated microstructure of the alloy NbSiTiHf-5Al-5Cr and EPMA X-ray maps corresponding to (**b**) of (**c**) Nb, (**d**) Si, (**e**) Ti, (**f**) Cr, (**g**) Al and (**h**) Hf.

The phases present in the heat treated alloys were the same as in the cast alloys (Figure S1 in Supplemental data). The microstructure of the alloy NbSiTiHf-5Al (Figure 2) was similar to that shown in Figure 1a. Prior eutectic areas could still be seen only in the bulk of the alloy NbSiTiHf-5Al where the Al+Si concentration was 22.4 at.%. There was no Hf rich Nb_ss_ in the alloy NbSiTiHf-5Al-5Cr but in both alloys the partitioning of Hf and Ti in Nb_5_Si_3_ had progressed further resulting in the formation of discrete Hf rich Nb_5_Si_3_ grains, particularly in the alloy NbSiTiHf-5Al-5Cr (see Figure 2). Precipitation of a second phase in the Nb_5_Si_3_ was not observed in both the heat treated alloys.

In the heat treated alloy NbSiTiHf-5Al, the Ti and Hf concentrations in the Hf rich Nb_5_Si_3_ were the same as in the cast alloy. However, in the “normal” Nb_5_Si_3_ in the bulk of the heat treated alloy the Ti concentration had increased compared with the cast alloy, as did the Hf concentration. In other words, as a result of the heat treatment the Nb_5_Si_3_ in the alloy NbSiTiHf-5Al tended to become richer in Ti and Hf. 

In the heat treated alloy NbSiTiHf-5Al-5Cr, the partitioning of Hf led to the formation of Hf rich Nb_5_Si_3_, Hf poor Nb_5_Si_3_, and “normal” Nb_5_Si_3_; each had different composition and was observed in different locations within the microstructure. The Hf rich phase formed as discrete grains, see Figure 2. The Hf poor Nb_5_Si_3_ was found in the bulk of Nb_5_Si_3_ owing to incomplete homogenisation and diffusion of Hf from the surrounding Nb_ss_.

The elemental maps in Figure 2 show the dispersion and partitioning of elements within the microstructure of the heat treated alloy NbSiTiHf-5Al-5Cr. In these maps the red areas have a higher concentration of the analysed element, and in the blue areas there is little or none of the analysed element. The Nb map in conjunction with the Hf map shows that in areas of low Nb, the Hf levels were higher. This relationship between Nb and Hf (see discussion), when compared with the Si map, is due to the partitioning effect of Hf within the Nb_5_Si_3_. Titanium can be seen to be partitioning primarily to the phases that are rich in Hf. The Cr concentration is predominantly higher in the Nb_ss_ and is very low in the Nb_5_Si_3_ and Hf-poor Nb_5_Si_3_ grains. As would be expected, the Cr concentration in the Hf-rich Nb_5_Si_3_ was slightly higher due to the affinity of Cr with Ti and Hf. The Al concentration was fairly constant throughout the alloy with a slightly higher concentration in the Nb_ss_ and Hf-rich Nb_5_Si_3_. In the Nb and Si maps (Figure 2c,d) the areas of lower Nb and higher Si show the “halo effect” around the outside of the Hf rich Nb_5_Si_3_ (see Figure 2c). This increase in Si and decrease in Nb may suggest that the Hf-rich Nb_5_Si_3_ was still growing and these boundary areas were representative of an intermediate growth stage which heralds the progression of the Nb_5_Si_3_/Nb_ss_ interface and the consumption of Nb_ss_.

In the Nb_ss_ the average Si concentration was reduced to a value that is consistent with other data in the literature for heat treated Nb-silicide based alloys, see Table 1 and Table 2 [25,26]. The partitioning of Hf in the microstructure as well as its consumption to form hafnia (HfO_2_) led to a drop in the overall Hf concentration within the Nb_ss_ in both alloys after the heat treatment (Table 1 and Table 2). However, in the bulk of the alloy NbSiTiHf-5Al the Ti concentrations in the Nb_ss_ varied between 14.7 and 24.1 at.%. 

There was contamination by nitrogen and oxygen near the top and bottom surfaces of the heat treated alloy NbSiTiHf-5Al where TiN and HfO_2_ were formed but not in the bulk. Hafnia was also observed in the heat treated alloy NbSiTiHf-5Al-5Cr. The data for the alloy NbSiTiHf-5Al would suggest that the synergy of Al and Hf was effective in decreasing the diffusivity of interstitials to the bulk of the alloy, in agreement with [30]. 

### 3.3. Oxidation at 800 °C

After 100 h oxidation in air at 800 °C both alloys did not suffer from catastrophic pest oxidation (Figure S2 in Supplemental data), but there were differences in their oxidation behaviour. The alloy NbSiTiHf-5Al had followed parabolic oxidation kinetics with k_p_ = 4 × 10^−10^ g^2^/cm^4^s and after 100 h its mass gain was 0.0114 g/cm^2^ (Figure S4a in Supplemental data) The alloy NbSiTiHf-5Al-5Cr had followed linear oxidation kinetics with k_l_ = 1 × 10^−8^ g/cm^2^s and had gained mass 0.005 g/cm^2^ (Figure S5a in Supplemental data) There was no spallation of the scale formed on the alloy NbSiTiHf-5Al, and no oxide powder was observed near the specimen. However, there was evidence of scale detachment from the edge of the NbSiTiHf-5Al specimen. The oxidised specimen of the alloy NbSiTiHf-5Al-5Cr was generally intact. The scale that was formed on the alloy NbSiTiHf-5Al-5Cr was very brittle and easily crumbled into a powder; there was also a very small amount of oxide powder around the specimen after the isothermal oxidation experiment, which indicated early stages of pest oxidation. The weight gain data (Figures S4a and S5a in Supplemental data) and the morphology of the oxidised specimen indicated that there was no breakaway oxidation. 

**Figure 3:**
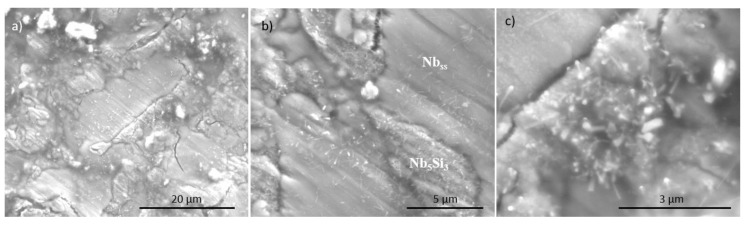
BSE images of scale on the surfaces of the alloy JN1 after isothermal oxidation at 800 °C, (**a**) showing overview of surface and (**b**) and (**c**) showing detail. The phases below the scale are indicated in (**b**).

Figure 3 shows images of the surface of the alloy NbSiTiHf-5Al-5Cr after oxidation at 800 °C. In Figure 3b the phases below the scale are indicated. The silicide can be clearly seen surrounded by oxidised Nb_ss_ (Figure 3a); the silicide itself was covered with fine needle-like oxide (Figure 3c). The Nb_5_Si_3_ displayed trans-granular and inter-granular cracking (Figure 3a) that was attributed to the strain induced by oxide growth [3,4] and the low toughness of Nb_5_Si_3_ [31].

**Figure 4:**
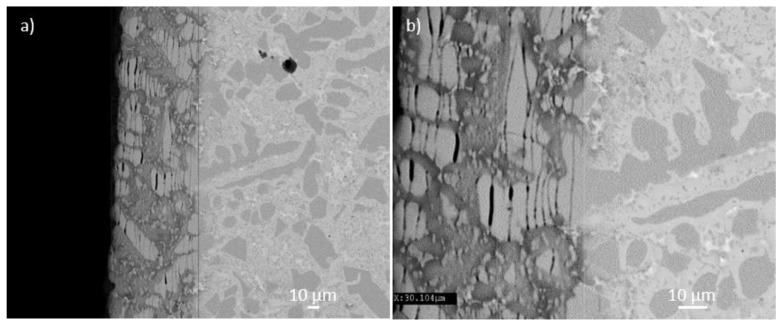
BSE image of cross sections of the alloy NbSiTiHf-5Al-5Cr after isothermal oxidation at 800 °C showing cracks in the diffusion zone. (**a**) overview, (**b**) detailed view.

Cross sections of the oxidised specimen of the alloys NbSiTiHf-5Al-5Cr and NbSiTiHf-5Al respectively are shown in Figure 4 and in Figure S6a in the Supplemental data. The diffusion zone [5] extended about 75 μm below the surface of the alloy NbSiTiHf-5Al-5Cr and there was cracking throughout the diffusion zone parallel to the oxide surface. This type of cracking is attributed to the voluminous oxide formation from the oxidation of the Nb_ss_, inducing tensile stresses in the low fracture toughness Nb_5_Si_3_ causing the latter to crack, and influences oxidation kinetics as reported before, for example see [4,5]. The cracks had travelled predominantly through the Nb_5_Si_3_ grains rather than the embrittled Nb_ss_ and oxides. The details of the microstructure shown in Figure 4 are essentially the same as those reported in [5] for the cast alloy JG4 (=Nb-24Ti-18Si-5Al-5Cr-5Hf-2Mo) after oxidation at 800 °C. The diffusion zone, in which the Nb_5_Si_3_ was cracked, comprised of Nb_5_Si_3_, heavily contaminated Nb_ss_, and a mixed oxide. The alloy below the diffusion zone was comprised of the same phases as the cast alloy before oxidation, however with oxygen contamination (Table S1 in Supplemental data). It should be noted that the phases were not contaminated by oxygen (EPMA data) in the bulk of the cast alloy. 

For the oxidised alloy NbSiTiHf-5Al-5Cr the EPMA analysis data was collected from the diffusion zone, 30 μm below the diffusion zone, and the bulk of the alloy in order to determine how deep the contamination by oxygen was. The diffusion zone was comprised of Nb_5_Si_3_ and a mixed oxide, the constituent phases of which are unknown. Compared with the cast alloy (Table 2) the Nb_5_Si_3_ showed little to no affinity for oxygen, with a low (0.4 at.%) concentration of oxygen in the diffusion zone and 0 at.% oxygen below the diffusion zone and in the bulk (Table S1 in Supplemental data). The latter is in agreement with the results reported in [5] for the alloy JG4 (=Nb-24Ti-18Si-5Al-5Cr-5Hf-2Mo). However, the Nb_ss_ in the alloy NbSiTiHf-5Al-5Cr had been contaminated and had an oxygen concentration of 1.4 at.% in the diffusion zone, which decreased towards the bulk of the alloy where the oxygen concentration was 0.8 at.% (Table S1 in Supplemental data). This confirmed that oxygen had contaminated (reached) the bulk of the alloy. The oxygen concentration in the Nb_ss_ was below the maximum solid solubility of oxygen in Nb_ss_ owing to the formation of HfO_2_ in the microstructure. Contamination of the Nb_ss_ and Nb_5_Si_3_ was observed in the diffusion zone and bulk of the alloy NbSiTiHf-5Al.

### 3.4. Oxidation at 1200 °C 

After 100 h oxidation in air at 1200 °C the alloy NbSiTiHf-5Al exhibited spallation of its scale (Figure S3a in Supplemental data). The spalled scale consisted of 12 layers uniformly separated after the oxidation; in other words, two layers had spalled-off from each side of the specimen. The layers could easily be crushed into powder after the oxidised specimen was removed from the crucible. There was also spallation of the scale that formed on the alloy NbSiTiHf-5Al-5Cr, however some parts of the scale remained attached, particularly near the edge of the specimen (Figure S3b in Supplemental data).

Powder XRD data (Figure S7b in Supplemental data) taken from the spalled layers of the scale formed on the alloy NbSiTiHf-5Al suggested that Ti niobates, HfO_2_, and Nb_2_O_5_, SiO_2_, and TiO_2_ [2] were the oxides in the scale (the same oxides as in the scale formed at 800 °C, Figure S7a in Supplemental data) and also indicated the presence of tetragonal and hexagonal Nb_5_Si_3_, which suggested that part of the spalled-off material included heavily contaminated substrate below the alloy scale. 

For both alloys there were no sudden weight gains or losses during oxidation (Figures S4b and S5b in Supplemental data); this indicated a stable oxide growth with no breakaway oxidation during isothermal oxidation. The mass gain of the alloy NbSiTiHf-5Al was 0.026 g/cm^2^, and the alloy followed parabolic kinetics in the early stages (<20 h) with k_p_ = 6 × 10^−8^ g^2^/cm^4^s and then linear kinetics with k_l_ = 1 × 10^−9^ g/cm^2^s (Figure S4b in Supplemental data). The mass gain of the alloy NbSiTiHf-5Al-5Cr was 0.05 g/cm^2^. The alloy followed parabolic oxidation kinetics with k_p_ = 7 × 10^−9^ g^2^/cm^4^s during the whole duration of the isothermal oxidation experiment (Figure S5b in Supplemental data).

**Figure 5:**
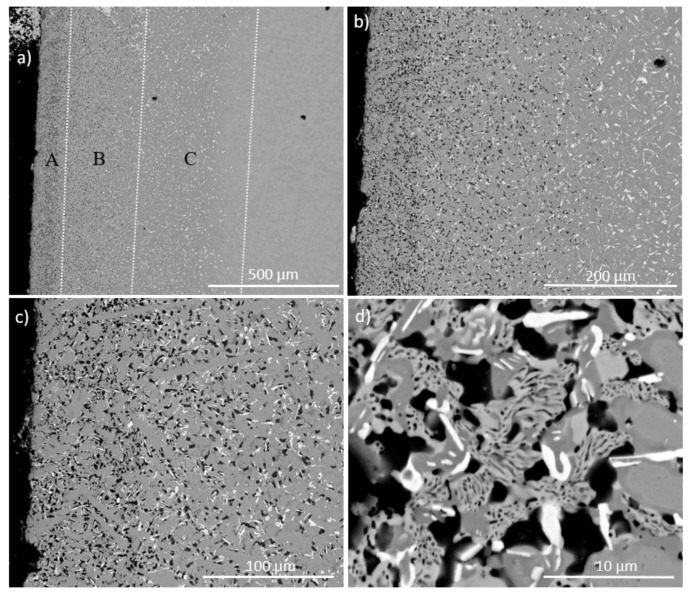
BSE images of cross section of the alloy NbSiTiHf-5Al-5Cr after isothermal oxidation at 1200 °C (**a**) showing overview of the sample and (**b**), (**c**) and (**d**) showing detail at greater magnification.

Unlike the alloy NbSiTiHf-5Al-5Cr oxidised at 800 °C, at 1200 °C the Nb_5_Si_3_ grains could no longer be clearly distinguished below the scale. The latter exhibited globular morphology, appeared to have some porosity and cracks and most likely consisted of Ti and Cr niobates, HfO_2_, and Nb_2_O_5_ [2,5]. Typical images of cross sections of the oxidised alloy NbSiTiHf-5Al-5Cr can be seen in the Figure 5, which shows the depth of the diffusion zone (about 600 µm, Figure 5a), different phases in the diffusion zone (Figure 5b,c), and details of the diffusion zone/oxide layer interface (Figure 5d). The diffusion zone consisted of three distinct regions. Closest to the oxide layer was region A, which was up to about 100 µm deep; this was a dense region consisting of oxides/nitrides and Nb_5_Si_3_ grains. Next was region B, which was about 100–350 µm deep and contained Nb_ss_, Nb_5_Si_3_, TiO/TiN (black contrast phase), and HfO_2_ (white contrast phase), and finally region C that was about 250–800 μm deep and consisted of Nb_ss_, Nb_5_Si_3_, and HfO_2_. The phases in region A could not be analysed using WDS, though similarities in contrast would suggest that it comprised of TiO/TiN and HfO_2_. There were several regions of different contrast which indicated the likely formation of a third oxide but it was not possible to analyse this phase. Due to the low Nb concentrations in the areas where titanium oxide(s) and/or nitride and HfO_2_ were formed, it is unlikely that a Nb-based oxide formed there. A cross section of the alloy NbSiTiHf-5Al is shown in Figure S6b in Supplemental data. There was spallation of the scales formed on both alloys, thus the presence or absence of cracking below the scale at 1200 °C cannot be confirmed. 

The chemical compositions of the phases in the bulk and the diffusion zone were analysed using WDS. The composition of Nb_5_Si_3_ was similar to that in the cast and heat treated alloy. Both the “normal” and Hf rich Nb_5_Si_3_ showed little affinity for oxygen, the maximum solubility of which was 0.7 at.% O. The Nb_ss_ close to the surface was contaminated by oxygen and in the diffusion zone its oxygen content was 3.3 at.% (Table S2 in Supplemental data). In the bulk of the sample, the Nb_ss_ was free of oxygen suggesting that the oxide formation at the surface and the scavenging of oxygen by Hf had stopped the oxygen ingress to the centre of the sample. The bright and black contrast regions (Figure 5) were analysed; the bright areas were HfO_2_ with a composition close to the stoichiometric ratio. The black precipitates were TiO_2_. Contamination of the Nb_ss_ and Nb_5_Si_3_ was observed in the bulk of the alloy NbSiTiHf-5Al which was more severe compared with 800 °C.

### 3.5. Hardness and Young’s Modulus

In the bulk of the heat treated alloy NbSiTiHf-5Al-5Cr the average nano-hardness values of the Nb_ss_ and Nb_5_Si_3_, respectively were 5.85 GPa (597 HV) and 17.4 GPa (1775 HV) and the Young’s moduli, respectively were 137.6 GPa and 241.4 GPa (see Table 3).

## 4. Discussion

### 4.1. Macrosegregation

There was macrosegregation of Si (MACSi) and Ti (MACTi) in both alloys, which was more severe in the alloy NbSiTiHf-5Al. Furthermore, the chemical inhomogeneity of these elements persisted after the heat treatment. 

**Figure 6:**
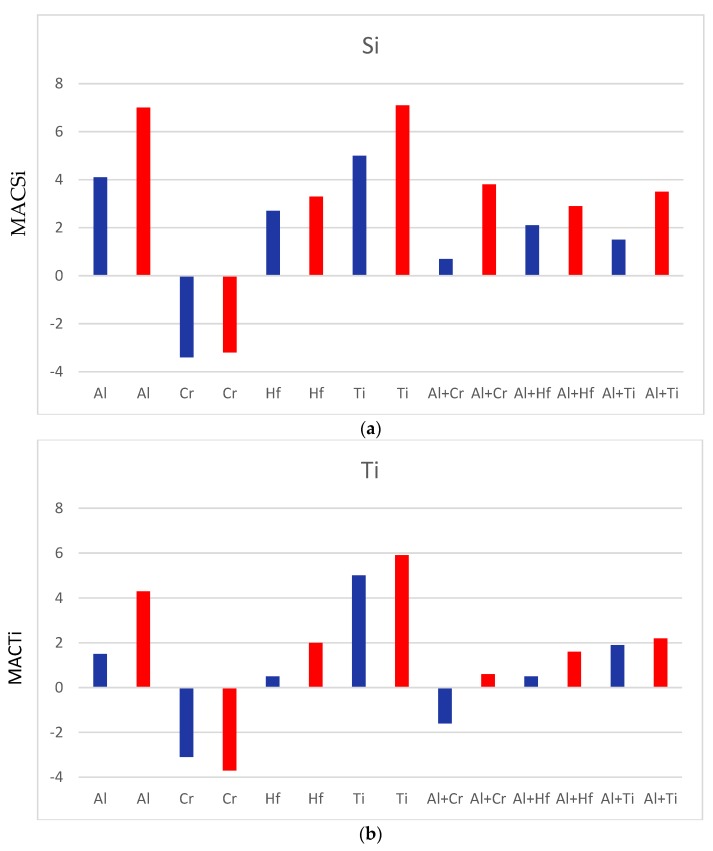
Effect of solute element(s) individually and in synergy on (ordinate) macrosegregation (at.%) of Si and Ti in cast microstructures (blue colour) and chemical inhomogeneity (at.%) of Si and Ti in heat treated microstructures (red colour). Data is shown as the difference from the values of the alloys NbSiTiHf-5Al and NbSiTiHf-5Al-5Cr, for example effect of Al on macrosegregation of Si MACSi_Al_ = MACSi^alloy^ ^NbSiTiHf-5Al^ − MACSi^alloy YG3^ = 7.4 − 3.3 = +4.1 at.%, and similarly MACSi_Cr_ = MACSi^alloy^ ^NbSiTiHf-5Al-5Cr^ − MACSi^alloy^ ^NbSiTiHf-5Al^ = 4 − 7.4 = −3.4 at.%. Alloy YG3 = Nb-18Si-5Hf-24Ti [26].

The effects of individual and simultaneous additions of specific elements on the macrosegregation after casting and on the chemical inhomogeneity after heat treatment of Si and Ti in Nb-silicide based alloys can be considered by comparing the data for different alloys. Figure 6 summarises the effects of Al, Cr, Hf, or Ti, and Al+Cr, Al+Hf, or Al+Ti on the macrosegregation after casting and chemical inhomogeneity after heat treatment of Si and Ti in Nb-silicide based alloys.

The nominal compositions of the alloys used for the comparisons are given below. When the effects of individual elements are considered, comparison of the alloys YG3 (=Nb-24Ti-18Si-5Hf [26]) and NbSiTiHf-5Al shows that Al increases MACSi and MACTi and that the chemical inhomogeneity of these elements persists after heat treatment. Similarly, comparison of the alloys NbSiTiHf-5Al and NbSiTiHf-5Al-5Cr shows that Cr decreases MACSi and MACTi, which is also supported by the data for the alloys KZ7 (=Nb-24Ti-18Si-5Al [25]) and KZ5 (=Nb-24Ti-18Si-5Al-5Cr [25]). The data for the alloys KZ5 and NbSiTiHf-5Al-5Cr, and KZ7 and NbSiTiHf-5Al show that Hf also increases MACSi and MACTi. Furthermore, comparison of the data for the alloys YG2 (=Nb-18Si-5Al-5Hf [26]) and NbSiTiHf-5Al shows that the addition of Ti increases MACSi. 

Regarding the effects of two alloying additions simultaneously, (i) comparison of the alloys YG3 and NbSiTiHf-5Al-5Cr shows that Al+Cr increase MACSi (Al eliminates the effect of Cr on MACSi) and decrease MACTi (Cr eliminates the effect of Al on MACTi), (ii) comparison of the alloys KZ4 (=Nb-24Ti-18Si-5Cr [25]) and NbSiTiHf-5Al-5Cr shows that Al+Hf increase MACSi and MACTi and that the synergy of these two elements reduces the effect of Al, and finally (iii) comparison of the alloys YG1 (=Nb-18Si-5Cr-5Hf [26]) and NbSiTiHf-5Al-5Cr shows that Al+Ti increase MACSi (the synergy of these two elements reduces the effect of each element individually on MACSi) and MACTi. 

The effect of the alloying additions of Al, Cr, Hf, or Ti on the macrosegregation of Si can also be studied using the parameters discussed in [29]. The macrosegregation of Si increases from the alloy KZ5 (MACSi = 1.3 at.%) to KZ4 (1.9 at.%) to KZ7 (2.3 at.%) to YG2 (2.4 at.%) to YG1 (2.5 at.%) to YG3 (3.3 at.%) to NbSiTiHf-5Al-5Cr (4 at.%) to NbSiTiHf-5Al (7.4 at.%) and this increase is accompanied by an increase of the parameters T_m_^sp^, ΔH_m_^sp^, ΔH_m_^alloy^/T_m_^alloy^ and a decrease of the parameter ΔH_m_^sd^/ΔH_m_^sp^ [29]. Details about the calculation of parameters are given in [29] but generally, T_m_^sp^ = Σx_i_T_mi_^sp^ where T_mi_ is melting temperature of sp element i, and x_i_ is at.% of element i; ΔH_m_^sp^ = Σx_i_ΔH_mi_^sp^ where ΔH_mi_ is enthalpy of melting of sp element i; ΔH_m_^alloy^/T_m_^alloy^ = Σx_i_ΔH_mi_^alloy^/Σx_i_T_mi_^alloy^ or the ‘alloy entropy of fusion’, and ΔH_m_^sd^/ΔH_m_^sp^ = ratio of enthalpy of melting of sd elements to the sp elements. The synergy of Al, Cr, and Ti (alloy KZ5) gives the lowest MACSi and the latter increases when Hf is simultaneously present with the aforementioned elements (alloy NbSiTiHf-5Al-5Cr). The strongest effect on MACSi is from the synergy of Al, Hf, and Ti (alloy NbSiTiHf-5Al).

### 4.2. Microstructure

In the cast alloy NbSiTiHf-5Al there was Hf (and Ti) rich Nb_5_Si_3_ in the very bottom of the button and in the “transition zone” from the bottom to the bulk, and “normal” Nb_5_Si_3_ in the latter and in the bulk and top of the button. Compared with the cast alloys KZ7 (=Nb-24Ti-18Si-5Al [25]) and YG3 (=Nb-24Ti-18Si-5Hf [26]), no Ti rich areas were observed in the Nb_ss_ in NbSiTiHf-5Al. However, in contrast to the alloy NbSiTiHf-5Al, no Ti and Hf rich Nb_5_Si_3_ was observed in the alloy YG3. In the alloy NbSiTiHf-5Al (and also in the alloy NbSiTiHf-5Al-5Cr) the Hf (and Ti) rich Nb_5_Si_3_ formed as distinct grains as did the “normal” Nb_5_Si_3_. In the very bottom of the button of the alloy NbSiTiHf-5Al the Nb_ss_ was leaner in Hf and Ti compared with the Nb_ss_ in the bulk and top. The above discussion would suggest that cooling rate and melt composition (both Hf and Al were present in NbSiTiHf-5Al compared with the alloys KZ7 (no Hf) and YG3 (no Al)), influenced the partitioning of Hf and Ti between the melt, Nb_5_Si_3_, and Nb_ss_. The presence of βNb_5_Si_3_ and no αNb_5_Si_3_ in the cast alloy NbSiTiHf-5Al is consistent with the effect the addition of Al has on the type of primary Nb_5_Si_3_ formed in the cast microstructure [5]. Comparison with the cast alloy YG3 would suggest that the synergy of Hf with Al strengthened the effect of Al, meaning it did not promote the βNb_5_Si_3_ → αNb_5_Si_3_ transformation during solid state cooling. 

The suppression of the L → Nb_ss_ + Nb_3_Si eutectic is consistent with the absence of Nb_3_Si in the cast alloy NbSiTiHf-5Al, which is known to be suppressed by Al (see data for the alloy KZ7 in [25] and the alloy YG3 in [26], in the latter (no Al) the above eutectic was formed). The average Si+Al composition (≈20.6 at.%) of the Nb_ss_+Nb_5_Si_3_ eutectic was in agreement with [27]. 

As the βNb_5_Si_3_ formed in the very bottom of the button of the alloy NbSiTiHf-5Al, the surrounding melt became lean in Si and Hf and rich in Ti and Al. In this melt, Nb_ss_ formed and the melt became rich in Si and Hf and poor in Al, and its Ti concentration did not change. As the cooling rate decreased, in the latter melt “normal” βNb_5_Si_3_ formed, the surrounding melt became lean in Si and Ti, and Hf had more time to partition to the melt, which thus became richer in Al, Hf, and Ti. From this melt Nb_ss_—slightly richer in Ti and Hf compared with the Nb_ss_—formed in the bottom of the button and as this Nb_ss_ formed the melt became rich in Si, Hf and poor in Al. When the Si+Al composition of the melt approached that of the Nb_ss_+Nb_5_Si_3_ eutectic the latter formed either in-between Nb_ss_ grains or from the Nb_ss_ that was formed on Nb_5_Si_3_ grains. Thus, from the observed microstructure in the cast alloy NbSiTiHf-5Al it is suggested that the solidification path in the bulk of the alloy NbSiTiHf-5Al was L → L+βNb_5_Si_3_ → L+βNb_5_Si_3_+Nb_ss_ → βNb_5_Si_3_+Nb_ss_+(βNb_5_Si_3_+Nb_ss_)_eutectic_ with hexagonal γNb_5_Si_3_ forming in the early stages of solidification (i.e., near the water cooled copper crucible), owing to the partitioning of Hf and Ti.

The formation of C14-NbCr_2_ in Nb-silicide alloys is linked with the partitioning of Cr to the melt which, as the solidification progresses, becomes rich in Cr and the Laves forms from the last to solidify Cr-rich melt [25]. In the cast alloy NbSiTiHf-5Al-5Cr the partitioning of Hf in the Nb_ss_ and βNb_5_Si_3_ and the corresponding increase in the concentration of Cr (and Ti) in the Hf (and Ti) rich Nb_ss_ starved the inter-dendritic melt from the high Cr concentration that would have promoted the formation of C14-NbCr_2_. Areas in the microstructure of the cast alloy NbSiTiHf-5Al-5Cr where Laves phase could form were much too small to analyse using EDS/WDS. The X-ray maps (Figure 1) were non-conclusive regarding the presence of the Laves phase. If the Laves phase was present in the alloy NbSiTiHf-5Al-5Cr, it is not likely that it had formed at a high volume fraction. 

The C14-NbCr_2_ Laves phase was formed in the cast alloy KZ5 (=Nb-24Ti-18Si-5Cr [25]). Another reason for the stabilisation of the C14-NbCr_2_ Laves phase in the cast alloy KZ5 and not in the alloy NbSiTiHf-5Al-5Cr would be the higher actual Cr concentration in KZ5, which was 6.8 to 7.6 at.% [25]. The Nb_5_Si_3_ grains and the eutectic were much coarser in the alloy KZ5, compared with the alloys NbSiTiHf-5Al and NbSiTiHf-5Al-5Cr; this would suggest a refining effect of Hf on the microstructure and is in agreement with [32]. 

From the observed microstructure in the cast alloy JN1, the following solidification path is suggested: L → L + βNb_5_Si_3_ → L + βNb_5_Si_3_ + Nb_ss_ → βNb_5_Si_3_ + Nb_ss_ + (βNb_5_Si_3_+Nb_ss_)_eutectic_. Regarding the structure of Nb_5_Si_3_, the XRD data for the cast alloy NbSiTiHf-5Al-5Cr confirmed only the presence of tetragonal βNb_5_Si_3_. The volume fractions were 0.42 and 0.58, respectively for the Nb_ss_ and the βNb_5_Si_3_.

The bcc Nb solid solution was stable in both the alloys NbSiTiHf-5Al and NbSiTiHf-5Al-5Cr and in the latter it also formed Hf rich regions, which were not observed after the heat treatment. Hafnium and Ti partition to the Nb_ss_ where the increase in the concentration of one of these elements is accompanied by an increase in the concentration of the other. In other words, “Ti and Hf like each other in the solid solution”. Thus, the Hf rich Nb_ss_ in the alloy NbSiTiHf-5Al-5Cr was also Ti rich compared with the “normal” Nb_ss_. Comparison of the data for the alloys YG3 (=Nb-24Ti-18Si-5Hf [26]) and NbSiTiHf-5Al shows that the synergy of Al with Hf in the latter alloy decreased the partitioning of Ti in the Nb_ss_ (Ti-rich Nb_ss_ was observed only in the cast alloy YG3). The increase of the Cr and Hf concentrations in the Hf rich Nb_ss_ in the alloy NbSiTiHf-5Al-5Cr was in agreement with the dependence of the concentrations of these elements on the Ti concentration in the Nb_ss_. 

**Figure 7:**
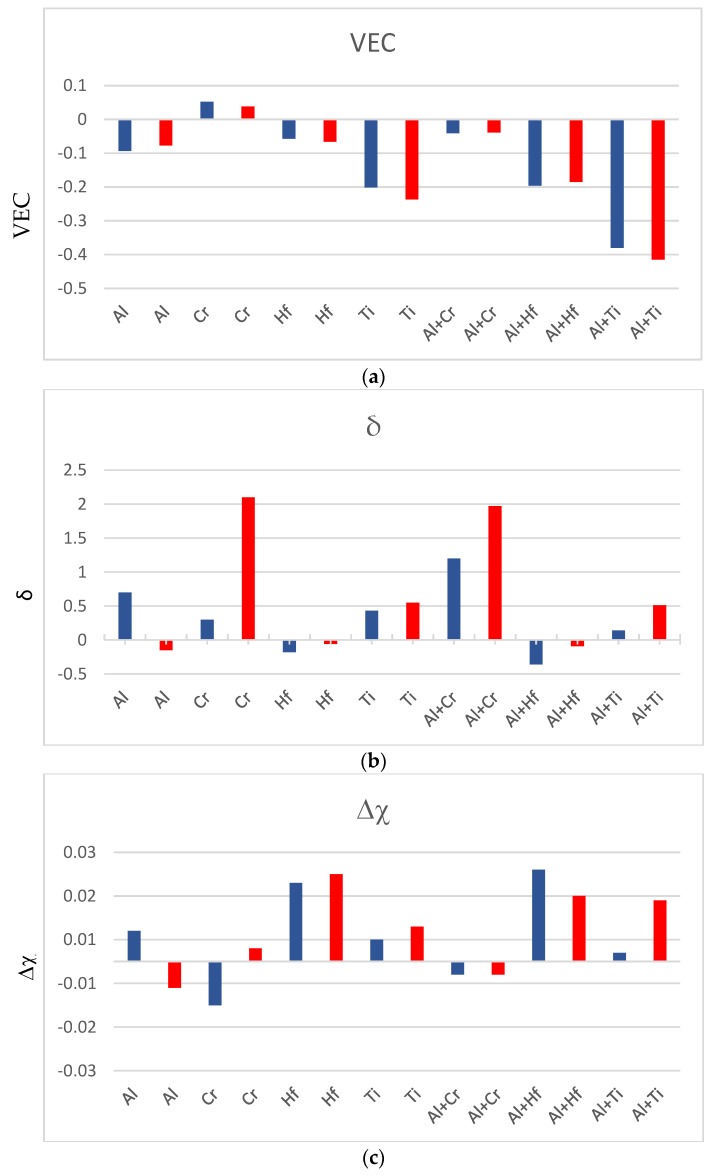
Effect of solute element(s) individually and in synergy on the parameters (ordinate) VEC, δ and Δχ of the Nb_ss_ in cast microstructures (blue colour) and in heat treated microstructures (red colour). Data is shown as the difference from the values of the alloys NbSiTiHf-5Al and NbSiTiHf-5Al-5Cr, for example, the effect of Al on the parameter VEC is VEC_Al_ = VEC^Nbss^ ^NbSiTiHf-5Al^ − VEC^Nbss YG3^ = 4.586 − 4.679 = −0.093, similarly δ_Al_ = δ^Nbss NbSiTiHf-5Al^ − δ^Nbss YG3^ = 4.34 − 3.64 = +0.7. The nominal composition of the alloy YG3 is Nb-24Ti-18Si-5Hf [26].

The parameters VEC (valence electron concentration), δ (related to atomic size), and Δχ (related to electronegativity) can describe the alloying behaviour of the bcc Nb_ss_ [33]. The effects of alloying additions individually and simultaneously in Nb-silicide based alloys on the alloying behaviour of their Nb solid solutions can be determined by comparing data for different alloys. For example, comparison of the alloys YG3 and NbSiTiHf-5Al shows that Al decreases VEC, and comparison of the alloys YG2 (=Nb-18Si-5Hf-5Al [26]) and NbSiTiHf-5Al shows that Ti decreases VEC, and increases δ and Δχ. The data is summarised in Figure 7, which shows the effects of Al, Cr, Hf, or Ti, and Al+Cr, Al+Hf, or Al+Ti on the parameters VEC, δ, and Δχ. The latter parameters were calculated as described in [33]. It should be noted that these three parameters are key for the design of Nb-silicide based alloys and their solid solutions, and also are linked with their oxidation and creep properties, see [2].

**Figure 8:**
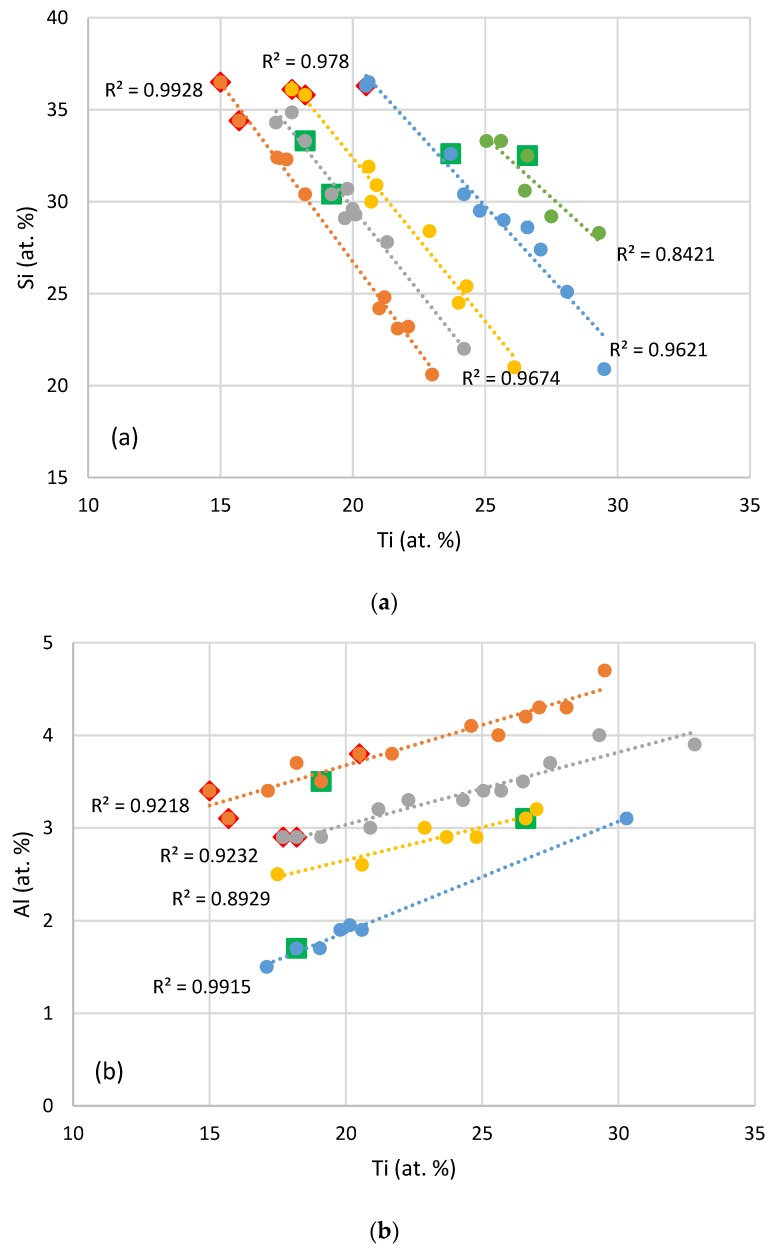

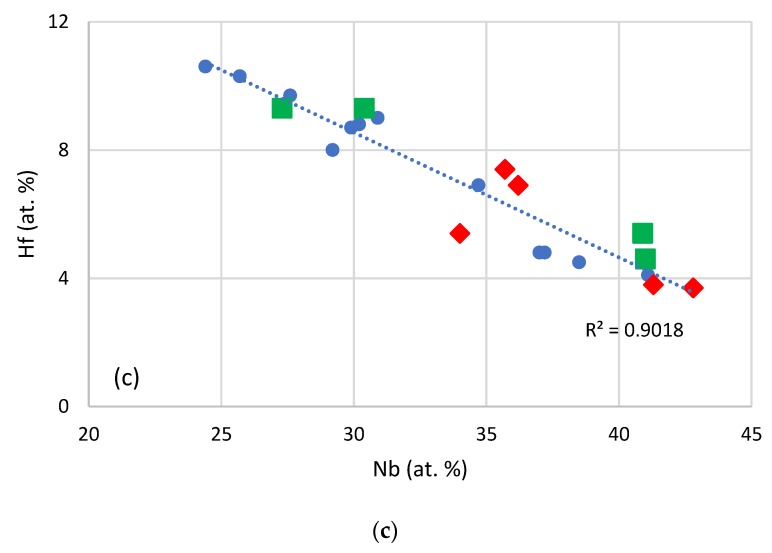
(**a**) Si versus Ti in Nb_5_Si_3_, (**b**) Al versus Ti in Nb_5_Si_3_, (**c**) Hf versus Nb in Nb_5_Si_3_. Squares and diamonds show data for the alloys NbSiTiHf-5Al-5Cr and NbSiTiHf-5Al, respectively. The data is for Nb_5_Si_3_ silicides in alloys studied in our research group (see [34] and [5,25]). In (**a**) series a (R^2^ = 0.9928) with alloying element additions of Al, Cr, Ge, Hf, Si, Sn, Ti; series b (R^2^ = 0.9674) Al, B, Cr, Ge, Hf, Mo, Si, Sn, Ta, Ti; series c (R^2^ = 0.978) Al, B, Cr, Ge, Hf, Si, Sn, Ta, Ti; series d (R^2^ = 0.9621) Al, B, Cr, Ge, Hf, Mo, Si, Ta, Ti; and series e (R^2^ = 0.8421) Al, B, Cr, Hf, Mo, Si, Sn, Ta, Ti. In (**b**) series a (R^2^ = 0.9218) with alloying element additions Al, B, Cr, Ge, Hf, Si, Sn, Ti; series b (R^2^ = 0.9232) Al, B, Cr, Ge, Mo, Si, Sn, Ta, Ti; series c (R^2^ = 0.8929) Al, B, Cr, Hf, Si, Sn, Ti; and series d (R^2^ = 0.9915) Al, B, Cr, Mo, Si, Sn, Ta, Ti. In (**c**) blue data points are for Nb_5_Si_3_ silicides with alloying element additions of Al, B, Cr, Ge, Hf, Si, Sn, Ti.

The Al, Hf, and Ti concentrations in the Nb_5_Si_3_ silicide are indicated in Figure 8, which shows the trends of the Si versus Ti, Al versus Ti, and Hf versus Nb concentrations in Nb_5_Si_3_ in Nb-silicide based alloys studied in our research group, see [34]. The alloying elements in each sub-set of data are given in the figure caption. The data in Figures 1 and 8 in [34] show that there exist strong correlations between solute elements in Nb_5_Si_3_. The data points for the Nb_5_Si_3_ in the alloys NbSiTiHf-5Al and NbSiTiHf-5Al-5Cr fall on the trends that have been found in our research group (for example see [5,25,34]). Figure 8a shows that the Si concentration in Nb_5_Si_3_ decreases with increasing Ti concentration. The Al concentration in Nb_5_Si_3_ exhibits the opposite trend with increasing Ti content (Figure 8b). The concentration of Hf in Nb_5_Si_3_ also increases with the Ti concentration (the data for Hf versus Ti in Hf rich and “normal” Nb_5_Si_3_ gives R^2^ = 0.77, figure not shown). Interestingly, the Hf concentration in the Nb_5_Si_3_ is also related with that of Nb, as shown in [34]. In Figure 8c the best linear fit of the data excluding the alloys NbSiTiHf-5Al-5Cr and NbSiTiHf-5Al gives R^2^ = 0.952. 

**Figure 9:**
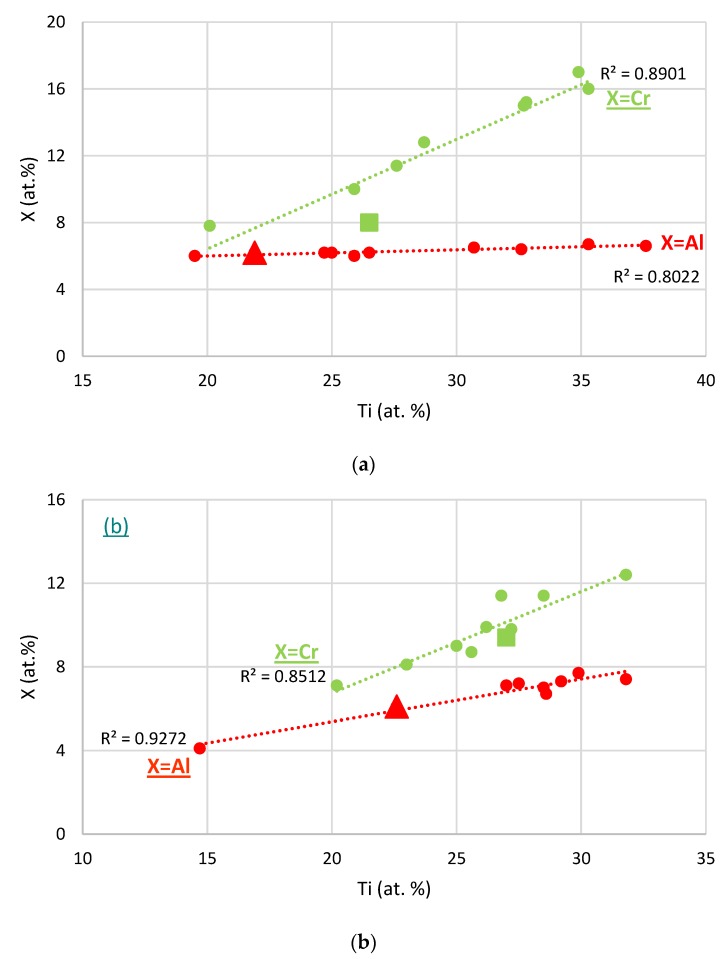
Data for solute element X=Al or Cr in Nb_ss_ (**a**) in cast and (**b**) heat treated KZ series alloys (data is shown by filled circles), and in the NbSiTiHf-5Al and NbSiTiHf-5Al-5Cr alloys. The data for X = Al or Cr in Nb_ss_ in NbSiTiHf-5Al-5Cr and NbSiTiHf-5Al is shown by squares and triangles, respectively. Data for X=Al or Cr are shown in red and green, respectively. The alloying elements in KZ series alloys are Al, Cr, Si, Ti, for alloy compositions see [25].

The relationships between the Al and Ti, or Cr and Ti concentrations in the Nb_ss_ in cast and heat treated KZ series alloys [25] are shown in Figure 9. It is seen that the concentrations of Al and Cr in the Nb_ss_ increase with its Ti concentration. The data for Al in the cast and heat treated alloys NbSiTiHf-5Al and NbSiTiHf-5Al-5Cr and for Cr in the heat treated alloy NbSiTiHf-5Al-5Cr falls on or is very close to the best linear fit lines in Figure 9. 

The microstructure of the heat treated alloy NbSiTiHf-5Al consisted of βNb_5_Si_3_ and γNb_5_Si_3_, Nb_ss_, and coarsened prior eutectic. Comparison with the heat treated alloys KZ7 (=Nb-24Ti-18Si-5Al [25]) and YG3 (=Nb-24Ti-18Si-5Hf [26]) would suggest (i) that the addition of Hf stabilised the βNb_5_Si_3_ and (ii) that the synergy of Al with Hf and Ti promoted the stability of γNb_5_Si_3_. No Nb_ss_ precipitates were observed in the Nb_5_Si_3_ in the heat treated alloys NbSiTiHf-5Al and NbSiTiHf-5Al-5Cr, which is consistent with the precipitation of Nb_ss_ often observed in αNb_5_Si_3_ as part of the βNb_5_Si_3_ to αNb_5_Si_3_ transformation in Nb silicide based alloys [25]. The presence of coarsened prior eutectic in the alloy NbSiTiHf-5Al compared with its absence in the alloys KZ7 and YG3 can be explained by the lower homologous heat treatment temperature. 

The current thermodynamic databases cannot account for the presence of bcc Nb_ss_, βNb_5_Si_3_, and hexagonal γNb_5_Si_3_ in the heat treated alloy NbSiTiHf-5Al. The alloy NbSiTiHf-5Al was heat treated at 1300 °C for 100 h, and it cannot be inferred that equilibrium was reached in its microstructure. In the alloy KZ7 (no Hf) the transformation βNb_5_Si_3_ → αNb_5_Si_3_ was completed after the 100 h heat treatment at 1500 °C. Thus, it is possible either that in the alloy NbSiTiHf-5Al the above transformation had not progressed enough (because of the lower heat treatment temperature) to give a volume fraction of αNb_5_Si_3_ that could be detected by XRD, or that the synergy of Hf and Al suppressed the above transformation. The latter is supported by the results for the heat treated (1500 °C/100 h) alloy NbSiTiHf-5Al-5Cr where the βNb5Si3 was still present, while in the heat treated (1500 °C/100 h) alloy KZ5 (=Nb-24Ti-18Si-5Al-5Cr [25]) both the βNb_5_Si_3_ and αNb_5_Si_3_ were present [25], and in both alloys (i.e., NbSiTiHf-5Al-5Cr and KZ5) the solubility of Cr in the Nb_5_Si_3_ was very low (Cr_5_Si_3_ and βNb_5_Si_3_ have W_5_Si_3_ as prototype and one would expect Cr in synergy with Al to stabilise the βNb_5_Si_3_). If we were to consider Nb, Hf, and Ti as equivalent in this alloy then the NbSiTiHf-5Al is in the Nb_ss_ + αNb_5_Si_3_ two phase area in the isothermal Nb-Si-Al sections at 1400 °C in [35] and 1300 °C in [36], and in the isothermal Ti-Al-Si sections at 1200 °C in [37] and 1270 °C in [38] the alloy falls in the bcc Ti_ss_ and Ti_5_Si_3_ two phase area. The latter would suggest that Hf and Ti, which form hexagonal Hf_5_Si_3_ and Ti_5_Si_3_ compounds with the same structure as the hexagonal γNb_5_Si_3_, promoted the stability of γNb_5_Si_3_ in the alloy NbSiTiHf-5Al. Therefore, the suppression of the βNb_5_Si_3_ to αNb_5_Si_3_ transformation in this alloy could be linked with the stability of the γNb_5_Si_3_. The latter would suggest that after prolonged heat treatment the γNb_5_Si_3_ could be stable in the alloy NbSiTiHf-5Al-5Cr. It should be noted that Hf rich Nb_ss_ was formed in the cast alloy NbSiTiHf-5Al-5Cr but not in the cast alloy NbSiTiHf-5Al, and therefore less Hf was available in the former alloy to partition to Nb_5_Si_3_. The diffusion distance of Hf in Nb is ≈18 µm and 31µm, respectively after 100 and 300 h at 1500 °C.

### 4.3. Hardness and Young’s Modulus

Data for the nano-hardness and micro-hardness and the Young’s moduli of Nb_ss_ and Nb_5_Si_3_ for heat treated alloys are compared in Table 3. The microhardness (mHV) is lower than the nano-hardness (nHV), in agreement with the literature [39]. For KZ type alloys [25] data from our research group gives mHV_Nbss_ = 0.7545 (nHV_Nbss_) and mHV_Nb5Si3_ = 0.633 (nHV_Nb5Si3_). It was not possible to measure the microhardness of the Nb_ss_ and Nb_5_Si_3_ in the heat treated alloy NbSiTiHf-5Al-5Cr (the different phases could not be distinguished) and the nano-hardness and micro-hardness of the same phases in the heat treated alloy NbSiTiHf-5Al. The values given in bold italics for Nb_ss_ and Nb_5_Si_3_ in Table 3 were calculated using the above relationships. The hardness data for the Nb_ss_ shows that the synergy of Al and Cr has a stronger hardening effect than that between Al, Cr, and Hf. The micro-hardness of binary (unalloyed) βNb_5_Si_3_ is 1360 HV [34]. The data in Table 3 shows that (Nb,Ti)_5_(Si,Al)_3_, (Nb,Ti,Cr)_5_(Si,Al)_3_, and (Nb,Ti,Cr,Hf)_5_(Si,Al)_3_ have hardness lower than 1360 HV and that the substitution of Nb only by Ti and of Si only by Al has the smallest negative effect on the hardness of Nb_5_Si_3_ (see [34]). 

The Young’s moduli of Nb, αNb_5_Si_3_, and βNb_5_Si_3_, respectively are 101.9, 291, and 269 GPa [40]. The data in Table 3 shows that the synergy of Al, Cr, and Ti had a stronger negative effect compared with the synergies between Al and Ti and between Al, Cr, Hf, and Ti. Furthermore, the data for the Nb_5_Si_3_ in Table 3 is in agreement with the calculations that showed higher modulus for binary (unalloyed) αNb_5_Si_3_ than βNb_5_Si_3_. Calculations in [40] for Nb_5_Si_3_ alloyed with Ti showed that when 12.5 at.% Ti substitutes Nb in the Nb_5_Si_3_, the moduli of α(Nb_50_Ti_12.5_)Si_37.5_ and β(Nb_50_Ti_12.5_)Si_37.5_ were 313.8 and 238.5 GPa, respectively. The data in Table 3 for the alloy KZ7 would suggest that the substitution of Nb by Ti, and Si by Al, reduced the modulus of αNb_5_Si_3_. The same was the case for the βNb_5_Si_3_ when Nb was substituted by Ti, Cr, and Hf and Si by Al (data for the alloy NbSiTiHf-5Al-5Cr). 

### 4.4. Oxidation

When the isothermal oxidation in air of the alloys NbSiTiHf-5Al and NbSiTiHf-5Al-5Cr at 800 °C and 1200 °C is compared with the alloys YG1, YG2, YG3, KZ4, KZ5, and KZ7, at both temperatures the weight gains of the alloys NbSiTiHf-5Al and NbSiTiHf-5Al-5Cr after 100 h oxidation were lower than the other alloys (Table S3 in Supplemental data where the nominal compositions of the aforementioned alloys are given).

At 800 °C the synergy of Al, Hf, and Ti was most effective in the alloy NbSiTiHf-5Al where pest oxidation was suppressed and a thin and adherent scale formed. The data for the alloy NbSiTiHf-5Al-5Cr would suggest that the addition of Cr had a negative effect (early stages of pest oxidation, see Section 3.3) even though the weight gain after 100 h was smaller than that of the alloy NbSiTiHf-5Al. This effect was attributed to Cr. Among the KZ series alloys [25] the alloy KZ7 exhibited the best isothermal oxidation in air at 800 °C and higher oxidation rates were observed in alloys where Al and Cr were present simultaneously. 

The mass gain of the alloy KZ5 (Al and Cr but no Hf) was about 0.030 g/cm^2^ after 85 h at 800 °C. The data for the alloy NbSiTiHf-5Al-5Cr showed that the addition of Hf reduced the weight gain significantly but did not change the oxidation kinetics. Comparison of the oxidised specimens of the alloys KZ5 and NbSiTiHf-5Al-5Cr showed that the synergy of Hf with Al, Cr, and Ti also did not improve the adherence of the scale. However, the vol.% of Nb_ss_ in the alloy KZ5 (about 52%) was higher than that in the alloy NbSiTiHf-5Al-5Cr (about 42%). The Nb_ss_ is known to have a negative effect on oxidation when its volume fraction increases in Nb-silicide based alloys [5]. 

At 800 °C the Nb_ss_ was contaminated by oxygen even in the bulk of both alloys, but the severity of contamination decreased with distance from the scale/substrate interface. The Nb_5_Si_3_ was also contaminated by oxygen but only in the diffusion zone and its contamination was less severe compared with the Nb_ss_.

At 1200 °C none of the alloys produced a stable spall resistant scale, the alloy NbSiTiHf-5Al gained the lowest weight among the alloys YG1, YG2, YG3, KZ4, KZ5, KZ7 (see Table S3 in Supplemental data for nominal compositions of alloys) and NbSiTiHf-5Al-5Cr, but the oxidation of the alloy NbSiTiHf-5Al-5Cr was considered better as improvement of scale adhesion was observed (Table S3 in Supplemental data). Alloying Nb-24Ti-18Si based alloys with Al, Cr, Hf individually (alloys KZ7, KZ4 and YG3) did not improve the adhesion of the scale which spalled off from all sides of the cube shaped oxidation specimens. The same spallation was observed when Al and Cr and Al and Hf were present simultaneously in the alloys KZ5 and NbSiTiHf-5Al, respectively. Evidence for improved scale adhesion was provided only by the alloy NbSiTiHf-5Al-5Cr, where Al, Cr, and Hf were in synergy with Ti. Improvement in scale adhesion with the addition of Sn has been reported in [5] for Nb-24Ti-18Si-5Al-5Cr-5Hf-5Sn-2Mo but not for MASC-xSn alloys in [41], where x = 2, 4, 5, 6, 8 (the nominal composition of the MASC alloy is Nb-25Ti-16Si-8Hf-2Al-2Cr, [1]). Alloy design/selection research in our group [2] has indicated that stable spall resistant scales can form with the addition of metalloid element in Nb-Si-TM-RM-Al based alloys [42,43,44], where TM = transition metal and RM = refractory metal. 

At 1200 °C the Nb_ss_ was not contaminated by oxygen in the bulk of the alloy NbSiTiHf-5Al-5Cr, but the severity of the contamination of the Nb_ss_ increased close to the scale/substrate interface (Table S2 in Supplemental data). The Nb_5_Si_3_ was also contaminated by oxygen more severely compared with the oxidation at 800 °C and its contamination was less severe compared with the Nb_ss_. The data for the alloy NbSiTiHf-5Al-5Cr would thus suggest that at 1200 °C the synergy of Al, Cr, and Hf with Ti also was effective for reducing the contamination of the bulk of the alloy by interstitials. This was attributed to the scavenging of oxygen by Hf and the reduced diffusivity of oxygen from the synergy of Al and Hf [30].

It is not possible to compare the cracking of the substrate just below the scales formed on the two alloys at 800 and 1200 °C because of the spallation of the thicker scales at the latter temperature (see Section 3.4). It is highly likely that at the higher temperature (i) the plasticity of the oxide(s) could be enhanced, (ii) there could be some plastic deformation of the substrate, and as oxidation progressed, (iii) the fracture behaviour of the Nb_ss_ and Nb_5_Si_3_ could vary with changes in the composition of the phases [31], and (iv) the mechanical properties of Nb_5_Si_3_ could also change with changing composition [40]. Thus, it is suggested that the deformation of the scale and substrate just below it could have been affected be one or more of (i) to (iv). 

In the oxides of the scales formed on both the alloys NbSiTiHf-5Al and NbSiTiHf-5Al-5Cr at 800 and 1200 °C the presence of Al niobate was not indicated by XRD (Figure S7 in the Supplemental data). The latter oxide was observed in the scale formed on the alloys KZ7 (=Nb-24Ti-18Si-5Al [25]) and KZ5 (=Nb-24Ti-18Si-5Al-5Cr [25]) [3] but not on the Hf containing alloy JG4 (=Nb-24Ti-18Si-5Al-5Cr-5Hf-2Mo [5]). This would suggest that the suppression of the formation of Al niobates in the scale of the alloys NbSiTiHf-5Al and NbSiTiHf-5Al-5Cr could be attributed to the addition of Hf. 

The oxidation of the single crystal Ni-based superalloy CMSX4 at 815 °C is parabolic with k_p_ = 4 × 10^−14^ g^2^cm^−4^s^−1^ and the weight gain is about 0.05 mg/cm^2^ after 50 h. At 1200 °C the oxidation also is parabolic with rate constant k_p_ = 4 × 10^−12^ g^2^cm^−4^s^−1^ and the weight gain is about 1 to 4 mg/cm^2^ after 50 h. The polycrystalline Nb-silicide based alloys NbSiTiHf-5Al and NbSiTiHf-5Al-5Cr did not match the oxidation of CMSX4.

## 5. Conclusions

In this paper the alloys Nb-24Ti-18Si-5Al-5Hf (alloy NbSiTiHf-5Al) and Nb-24Ti-18Si-5Al-5Cr-5Hf (alloy NbSiTiHf-5Al-5Cr) were studied in the as-cast and heat-treated conditions and after isothermal oxidation for 100 h at 800 and 1200 °C. The alloys were compared with alloys without Hf addition. The conclusions of the research are as follows:

1. In both alloys there was macrosegregation of Si (MACSi) and Ti (MACTi). The simultaneous addition of Al, Hf, and Ti in the alloy NbSiTiHf-5Al resulted in the highest macrosegregation of Si.

2. In both alloys the Nb_ss_ + βNb_5_Si_3_ eutectic was formed in the parts of the buttons where the melt had not solidified under the highest cooling rates. 

3. The βNb_5_Si_3_ and γNb_5_Si_3_ were formed in the cast alloy NbSiTiHf-5Al and only the βNb_5_Si_3_ in the cast alloy NbSiTiHf-5Al-5Cr. The same compounds were present in the heat treated microstructures of the two alloys where separate Hf rich Nb_5_Si_3_ grains formed, particularly in the alloy NbSiTiHf-5Al-5Cr. 

4. The Nb_ss_ was stable in both alloys. Hafnium and Ti rich Nb_ss_ was formed only in the cast alloy NbSiTiHf-5Al-5Cr. 

5. The Si and Al concentrations in Nb_5_Si_3_ respectively decreased and increased with increasing Ti concentration. The Hf concentration in the Nb_5_Si_3_ exhibited a stronger relationship with Nb than Ti.

6. The synergy of Al and Cr had a stronger hardening effect in the Nb_ss_ than that between Al, Cr, and Hf. The synergy of Al, Cr, and Ti had a stronger negative effect on the Young’s modulus of the Nb_ss_ compared with that of Al, Cr, Hf, and Ti. When Nb was substituted by Ti, Cr, and Hf and Si by Al the Young’s modulus of the βNb_5_Si_3_ was reduced compared with the unalloyed silicide. 

7. After 100 h at 800 °C both alloys did not suffer from catastrophic pest oxidation. Contamination of the Nb_ss_ and Nb_5_Si_3_ by oxygen was observed in both alloys, with that of the former phase being more severe. The Nb_5_Si_3_ was contaminated only in the diffusion zone. The Nb_ss_ was contaminated in the bulk of both alloys. 

8. At 1200 °C there was spallation of the scale formed on both alloys, which was more severe for the alloy NbSiTiHf-5Al. In both alloys the contamination of Nb_5_Si_3_ and Nb_ss_ by oxygen was increased slightly compared with 800 °C, and the silicide was not contaminated by oxygen in the bulk. The Nb_ss_ was not contaminated by oxygen only in the bulk of the alloy NbSiTiHf-5Al-5Cr.

## Figures and Tables

**Table 1:** SEM-EDS data (at.%) for the as cast and heat treated alloy Nb-24Ti-18Si-5Al-5Hf (average value, standard deviation, minimum and maximum analysis values).

Alloy & Phase	Nb	Si	Ti	Al	Hf
**As Cast**					
Average alloy	46.2 ± 2.1 42.1–49.8	21.3 ± 1.8 18.8–26.2	22.3 ± 1.2 20.1–25.3	4.5 ± 0.4 3.5–5.2	5.4 ± 0.4 4.5–6.0
Nb_ss_	64.8 ± 2.9 58.9–68.6	3.8 ± 1.2 2.5–5.4	21.9 ± 1.6 19.5–25.7	6.2 ± 0.2 6.0–6.7	3.3 ± 0.3 2.9–4.0
Nb_5_Si_3_	41.3 ± 1.2 40.5–44.5	36.5 ± 1.4 34.1–38.5	15.0 ± 0.3 14.0–15.5	3.4 ± 0.3 3.0–3.9	3.8 ± 0.3 3.6–4.4
Hf rich Nb_5_Si_3_	36.2 ± 2.2 31.5–38.1	36.1 ± 1.5 32.7–37.5	17.7 ± 3.0 15.9–24.7	2.9 ± 0.4 2.5–3.8	6.9 ± 0.5 6.5–7.8
Nb_ss_ + βNb_5_Si_3_ eutectic	52.6 ± 2.4 49.3–57.8	15.5 ± 1.2 12.1–17.9	22.1 ± 2.1 18.5–26.1	5.1 ± 0.4 4.5–5.7	4.6 ± 0.3 3.9–5.2
**Heat Treated**					
Average alloy	48.0 ± 1.9 44.5–51.6	20.5 ± 2.6 16.3–25.4	21.3 ± 1.2 19.2–25.1	4.3 ± 0.4 3.4–5.1	5.7 ± 0.3 5.1–6.4
Nb_ss_	68.2 ± 2.5 65.8–75.5	0.9 ± 0.3 0.4–1.5	22.6 ± 2.5 14.7–24.1	6.1 ± 0.5 4.1–7.0	2.1 ± 0.3 1.4–2.6
Hf rich Nb_5_Si_3_ (bulk *)	34.0 ± 1.1 33.2–34.8	36.3 ± 1.4 35.2–37.3	20.5 ± 0.6 20.1–20.9	3.8 ± 0.1 3.8–3.9	5.4 ± 0.1 5.3–5.5
Nb_5_Si_3_	42.8 ± 1.0 41.3–43.9	34.4 ± 0.6 33.5–35.1	15.7 ± 1.2 14.4–17.5	3.1 ± 0.4 2.5–3.6	3.7 ± 0.3 3.3–4.2
Hf rich Nb_5_Si_3_ (bottom *)	35.7 ± 3.1 27.9–38.6	35.8 ± 2.1 30.7–38.0	18.2 ± 3.1 14.5–24.6	2.9 ± 0.6 2.0–4.1	7.4 ± 0.7 6.3–8.6
Prior eutectic	48.7 ± 1.4 46.2–51.7	17.7 ± 1.2 15.2–19.4	23.4 ± 0.6 22.4–23.9	4.7 ± 0.3 4.4–5.1	5.5 ± 0.2 5.2–5.9

* see text.

**Table 2:** EPMA data (at.%) for the as cast and heat treated alloy Nb-24Ti-18Si-5Al-5Cr-5Hf (average value, standard deviation, minimum and maximum analysis values).

Alloy & Phase	Nb	Si	Ti	Cr	Al	Hf
**As Cast**						
Average alloy	41.2 ± 0.6 40.4–42.4	20.1 ± 1.0 17.7–21.7	23.5 ± 0.5 22.6–24.5	4.5 ± 0.3 4.0–5.0	5.5 ± 0.2 5.0–5.9	5.2 ± 0.2 5.0–5.6
Nb_ss_	53.7 ± 3.2 48.7–57.0	1.7 ± 0.4 1.3–2.5	26.5 ± 1. 924.7–29.5	8.0 ± 1.1 6.7–9.3	6.2 ± 0.2 6.2–6.8	3.6 ± 0.7 3.0–4.5
Hf rich Nb_ss_	45.9 ± 4.0 40.6–51.8	2.0 ± 0.7 1.3–3.5	30.7 ± 1.7 28.1–33.4	9.4 ± 1.0 7.5–11.1	6.5 ± 0.2 6.2–6.8	5.5 ± 0.7 4.7–6.4
Nb_5_Si_3_	41.0 ± 1.4 38.8–42.6	30.4 ± 1.2 28.2–31.5	19.2 ± 1.4 17.8–20.8	1.3 ± 0.5 0.9–2.3	3.5 ± 0.5 3.0–4.2	4.6 ± 0.1 4.4–4.7
Hf rich Nb_5_Si_3_	30.4 ± 0.6 30.1–31.3	32.6 ± 0. 931.2–33.3	23.7 ± 0.1 23.5–23.7	1.1 ± 0.4 0.9–1.8	2.9 ± 0.1 2.8–3.0	9.3 ± 0.3 8.9–9.5
Nb_ss_ + βNb_5_Si_3_ eutectic	46.8 ± 2.2 44.1–49.2	13.3 ± 1.5 12.0–14.9	24.2 ± 1.1 23.2–25.5	5.3 ± 0.6 4.8–6.1	5.6 ± 0.2 5.2–6.0	4.8 ± 0.5 4.6–4.9
**Heat Treated**						
Average alloy	43.1 ± 1.0 42.2–44.7	19.3 ± 1.8 15.3–21.2	24.1 ± 0.8 22.9–25.1	4.1 ± 0.5 3.5–5.0	3.8 ± 0. 92.5–4.9	5.7 ± 0.3 5.3–6.0
Nb_ss_	53.7 ± 0.8 53.1–54.9	0.5 ± 0.1 0.5–0.8	27.0 ± 0.1 26.9–27.2	9.4 ± 0.3 9.0–9.8	7.1 ± 0.7 6.1–7.7	2.2 2.1–2.2
Nb_5_Si_3_	40.9 ± 0.3 40.6–41.2	33.3 ± 0.2 33.1–33.6	18.2 ± 0.2 17.9–18.5	0.5 0.4–0.5	1.7 ± 0.1 1.6–1.8	5.4 ± 0.2 5.2–5.7
Hf rich * Nb_5_Si_3_ *	27.3 ± 0.3 27.1–27.9	32.5 ± 0.1 32.3–32.7	26.6 ± 0.1 26.3–26.7	1.2 1.2–1.3	3.1 ± 0.2 2.7–3.3	9.3 ± 0.1 9.0–9.5
Hf poor * Nb_5_Si_3_ *	41.3 ± 0.1 41.2–41.5	32.0 ± 0.3 31.4–32.4	18.8 ± 0.1 18.7–18.8	0.6 0.5–0.7	2.7 ± 0.3 2.4–3.2	4.6 ± 0.1 4.5–4.7

* see text.

**Table 3:** Hardness * and Young’s moduli of Nb_ss_ and Nb_5_Si_3_ in heat treated Nb-silicide based alloys.

Alloy	Phase	Micro-Hardness (Vickers)	Nano-Hardness (Vickers)	Nano-Hardness (GPa)	Young’s Modulus (GPa)
KZ7-HT	Nb_ss_	395.0 ± 19.0	502.0 ± 16.9	4.95	138.0 ± 12.0
KZ5-HT	Nb_ss_	466.0 ± 20.0	645.0 ± 31.2	6.5	131.0 ± 18.0
JN1-HT	Nb_ss_	450.0 *	597.0 ± 35.8	5.85	137.6 ± 10.4
KZ7-HT	αNb_5_Si_3_	1136.0 ± 60.0	1907.0 ± 106.0	18.7	283.8 ± 25.2
KZ5-HT	Nb_5_Si_3_	1131.0 ± 54.0	1685.0 ± 142.0	16.5	238.5 ± 23.6
JN1-HT	βNb_5_Si_3_	1124.0 *	1775.0 ± 153.0	17.4	241.4 ± 24.7

* see text.

KZ5 = Nb-24Ti-18Si-5Al-5Cr [25], KZ7 = Nb-24Ti-18Si-5Al [25], JN1 = NbSiTiHf-5Al-5Cr.

## Supplementary Materials

The following are available online at http://www.mdpi.com/1996-1944/11/9/1579/s1, Table S1: EPMA analysis data (at.%) for phases in the alloy NbSiTiHf-5Al-5Cr after isothermal oxidation at 800 °C, Table S2: EPMA analysis data (at.%) for phases in the alloy NbSiTiHf-5Al-5Cr after isothermal oxidation at 1200 °C, Table S3: Comparison of the isothermal oxidation of the alloys NbSiTiHf-5Al and NbSiTiHf-5Al-5Cr with other Nb silicide based alloys, Figure S1: X-ray diffractograms of the (a) as cast and (b) heat treated alloy NbSiTiHf-5Al and (c) cast and (d) heat treated alloy NbSiTiHf-5Al-5Cr. The non-indexed peaks correspond to un-identified phase(s), Figure S2: Specimens of the alloys NbSiTiHf-5Al (a) and NbSiTiHf-5Al-5Cr (b) after 100 h isothermal oxidation at 800 °C, Figure S3: Specimens of the alloys NbSiTiHf-5Al (a) and NbSiTiHf-5Al-5Cr (b) after 100 h isothermal oxidation at 1200 °C, Figure S4: Isothermal oxidation data for the alloy NbSiTiHf-5Al (a) 800 °C, (b) 1200 °C, Figure S5: Isothermal oxidation data for the alloy NbSiTiHf-5Al-5Cr (a) 800 °C, (b) 1200 °C, Figure S6: BSE images of cross sections of the alloy NbSiTiHf-5Al after isothermal oxidation (a) at 800 °C and (b) at 1200 °C, Figure S7: XRD data for the alloy NbSiTiHf-5Al (a) glancing angle data (GXRD, θ = 5 degrees) for 800 °C, (b) powder XRD data from the spalled off scale at 1200 °C.
