# Supplementary material for: A Study of the Effects of Al, Cr, Hf, and Ti Additions on the Microstructure and Oxidation of Nb-24Ti-18Si Silicide Based Alloys
Source: Materials (Basel). 2018 Sep 1;11(9):1579. doi: 10.3390/ma11091579 (PMC6164265; doi:10.3390/ma11091579)
Supplement: Supplementary file 1 [file materials-11-01579-s001.pdf]

materials

A Study of the Effects of Al, Cr, Hf, and Ti Additions on the Microstructure and Oxidation of Nb-24Ti-18Si Silicide Based Alloys

Jack Nelson, Mohammad Ghadyani, Claire Utton and Panos Tsakiropoulos *

Department of Materials Science and Engineering, Sir Robert Hadfield Building, The University of Sheffield, Mappin Street, Sheffield S1 3JD, UK; pha05jn@gmail.com (J.N.); m.ghadyani@sheffield.ac.uk (M.G.); c.utton@sheffield.ac.uk (C.U.)

* Correspondence: p.tsakiropoulos@sheffield.ac.uk

Examples of Hf containing Nb-silicide based alloys with transition, refractory, simple metal and metalloid additions (at.%)

Nb-22.0Ti-16.0Si-7.0Cr-3.0Al-2.0Hf-3.0Ta-0.1Ho → [1]

Nb-22.0Ti-16.0Si-6.0Cr-3.0Al-4.0Hf-1.5B-0.06Y → [2]

Nb-19.9Ti-19.7Si-9.9Cr-3.3Al-4.2Hf-4.2Ge → [3]

Nb-(14-25.5)Ti-(12-20)Si-(10-13)Cr-2Al-(2-4)Hf-X where X=B, Fe, Ge, Mo, Sn, Ta, W → [4]

Nb-(28.9-29.5)Ti-(7.7-8.4)Si-(10-10.4)Cr-(10-10.2)Al-(3-3.7)Hf-(3.4-4)Zr-(0-1.3)W-(0.9-1.3)Sn → [5]

Nb-(24.3-26.2)Ti-(11.9-17.6)Si-(2.5-6.7)Cr-(1.8-1.9)Al-(1.4-2)Hf-(0.2-5.5)Ge-(0.6-2.8)Fe-(0.3-1.6)Sn-(0.02-0.1)Ce-(0.2-2.3)B → [6]

References

1. Tian, Y.X.; Chen, G.G.; Guo, J.T.; Zhou, L.Z.; He, L.L.; Ye, He.Q. Microstructure and mechanical properties of directionally solidified Nb-22Ti-16Si-7Cr-3Al-3Ta-2Hf-0.1Ho alloy. *Adv. Eng. Mater.* **2007**, *9*, 963–966.
2. Guo, H.S.; Guo, X.P. Microstructure evolution and room temperature fracture toughness of an integrally directionally solidified Nb-Ti-Si based ultrahigh temperature alloy. *Scr. Mater.* **2011**, *64*, 637–640.
3. Menon, E.S.K.; Mendiratta, M.G.; Dimiduk, D.M. *Oxidation behaviour of complex niobium based alloys, in Niobium Science and Technology*; Niobium 2001 Ltd: Bridgeville, PA, USA, 2001; pp. 121–145.
4. Jackson, M.R.; Bewlay, B.P.; Zhao, J.-C. Niobium-Silicide Based Composites Resistant to High Temperature Oxidation. U.S. Patent 6,913,655 B2, 5 July 2005.
5. Tewari, R.; Song, H.-J.; Vasudevan, V.K.; Chatterjee, A. Microstructural characterisation of multicomponent Nb-Ti-Si-Cr-Al-X alloys. *Metall. Mater. Trans.* **2006**, *37*, 2669–2682.
6. Menon, E.S.K.; Mendiratta, M.G.; Dimiduk, D.M. High temperature oxidation mechanisms in Nb-silicide bearing multicomponent alloys. *Struct. Intermetallics* 2001, 591–600.

Table S1. EPMA analysis data (at.%) for phases in the alloy NbSiTiHf-5Al-5Cr after isothermal oxidation at 800 °C.

Area of interest	Phase	Nb	Si	Ti	Cr	Al	Hf	O
DZ*	Nb_5Si_3	39.1 ± 0.9<br>37.8–39.9	35.3 ± 1.2<br>33.8–36.8	17.2 ± 0.5<br>16.6–17.9	0.9 ± 0.1<br>0.8–1.0	2.8 ± 0.3<br>2.5–3.2	4.3 ± 0.1<br>4.2–4.4	0.4<br>0–2.0
	Mixed oxide	34.3 ± 1.2<br>33.4–36.0	0.8 ± 0.4<br>0.4–1.2	17.8 ± 0.6<br>16.8–18.1	4.6 ± 0.3<br>4.4–5.0	3.9 ± 0.3<br>3.6–4.2	2.8 ± 0.1<br>2.7–2.9	35.9 ± 1.7<br>33.6–37.9
30 μm below DZ*	Nb_{ss}	50.2 ± 3.5<br>46.9–54.2	1.7 ± 1.5<br>0.5–3.9	28.4 ± 1.5<br>26.6–29.9	8.1 ± 2.4<br>6.6–11.6	5.7 ± 0.2<br>5.5–6.0	4.4 ± 0.5<br>4.0–5.1	1.4<br>0–2.9
	Nb_5si_3	40.7 ± 0.2<br>40.4–40.9	34.2<br>34.2–34.2	17.4<br>17.4–17.4	0.8<br>0.8–0.9	2.6 ± 0.1<br>2.5–2.7	4.3<br>4.2–4.4	0
Bulk of specimen	Nb_{ss}	50.6 ± 1.8<br>49.0–52.5	1.7 ± 1.3<br>0.8–3.1	29.0 ± 0.7<br>28.6–29.9	8.1 ± 2.5<br>5.7–10.7	6.2 ± 0.2<br>6.0–6.4	3.5 ± 0.4<br>3.1–3.9	0.8<br>0–2.4
	Nb_5si_3	40.5<br>40.4–40.5	33.7<br>33.6–33.8	17.6<br>17.6–17.6	0.9<br>0.9–1.0	2.9 ± 0.1<br>2.9–3.0	4.3<br>4.3–4.4	0

*DZ = diffusion zone.

Table S2. EPMA analysis data (at.%) for phases in the alloy NbSiTiHf-5Al-5Cr after isothermal oxidation at 1200 °C.

Phase	Element						
	Nb	Si	Ti	Cr	Al	Hf	O
Nb_5Si_3 in DZ*	40.3 ± 0.9<br>38.5–41.8	32.1 ± 1.9<br>29.5–34.2	18.6 ± 0.9<br>17.8–19.8	0.7 ± 0.3<br>0.3–1.2	3.1 ± 0.3<br>2.4–3.5	4.5 ± 0.1<br>4.3–4.7	0.7<br>0–2.2
Hf rich Nb_5Si_3 in DZ*	25.2 ± 2.4<br>22.2–28.2	29.3 ± 2.6<br>25.7–32.8	27.8 ± 1.4<br>26.2–29.4	2.0 ± 0.8<br>0.9–2.9	3.7 ± 0.4<br>3.2–4.2	11.9 ± 1.1<br>10.5–13.5	0.1
Nb_{ss} in DZ*	45.3 ± 0.7<br>44.7–46.1	0.3	32.6 ± 0.5<br>32.1–33.1	10.5 ± 0.2<br>10.3–10.8	7.7 ± 0.1<br>7.5–7.7	0.2<br>0.2–0.3	3.3 ± 1.5<br>1.8–4.9
HfO_2	0.7<br>0.1–2.7	0	1.3 ± 0.9<br>0.5–2.7	0.3<br>0.2–0.6	0	37.7 ± 1.0<br>36.0–38.5	60.0 ± 1.9<br>56.7–61.3
Ti oxide	3.9 ± 2.8<br>1.9–5.9	0	47.7 ± 1.5<br>46.6–48.8	0.2<br>0.1–0.3	0.3<br>0.2–0.4	0.1	47.8 ± 4.3<br>44.7–50.8
Nb_{ss} in bulk of specimen	52.2 ± 0.1<br>52.1–52.3	0.3	29.5<br>29.5–29.4	9.2<br>9.2–9.3	6.9<br>6.8–6.9	1.9<br>1.9–1.9	0

*DZ=Diffusion zone.

Table S3. Comparison of the isothermal oxidation of the alloys NbSiTiHf-5Al and NbSiTiHf-5Al-5Cr with other Nb silicide based alloys.

Alloy	Temperature of isothermal oxidation					
	800 °C			1200 °C		
	ΔW time	Pest scale	Kinetics	ΔW time	Scale	Kinetics
YG1+	44 (18 h)	Yes Solid cube + powder	Linear	477 (26 h)	Maltese cross	Parabolic + linear
YG2+	163 (18 h)	Yes Solid pieces + powder	Linear	187 (96 h)	Maltese cross	Parabolic + linear
YG3+	52 (18 h)	Yes Solid cube + powder	Linear	31 (46 h)	Cube + scale spallation - all sides	Parabolic + linear
KZ4+	84 (85 h)	Maltese cross**	Linear	139 (65 h)	Cube + scale spallation – all sides	Parabolic + linear
KZ7+	20 (85 h)	No** Scale cracked along edges	Linear	101 (65 h)	Cube + scale spallation – all sides	Parabolic + linear
KZ5+	30 (85 h)	No** Scale cracked along edges	Linear	64 (65 h)	Cube + scale spallation – all sides	Linear
MG1+	11 (100 h)	No Scale cracked along edges	Parabolic	26 (100 h)	Cube + scale spallation - all sides	Parabolic + linear
JN1+	5 (100 h)	No* Scale cracked along edges	Linear	50 (100 h)	Cube + scale partial spallation	Parabolic

ΔW = weight gain (mg/cm^2).

* see text, ** see [3], + see text.

+ YG1=Nb-18Si-5Hf-5Cr [26], YG2=Nb-18Si-5Hf-5Al [26], YG3=Nb-18Si-5Hf-24Ti [26], KZ4=Nb-24Ti-18Si-5Cr [25],

KZ7=Nb-24Ti-18Si-5Al [25], KZ5=Nb-24Ti-18Si-5Al-5Cr [25], MG1=NbSiTiHf-5Al, JN1=NbSiTiHf-5Al-5Cr.

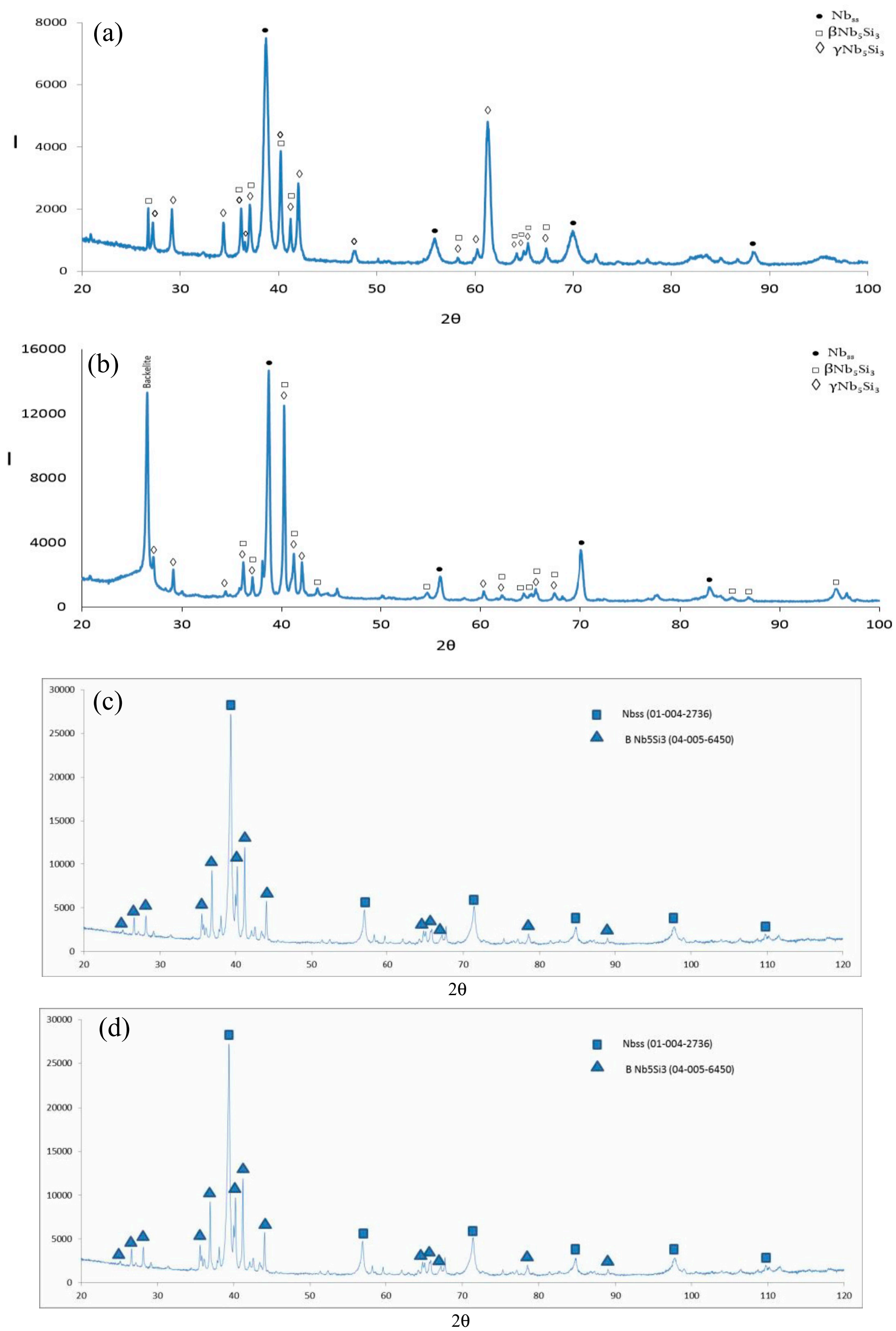

Figure S1. X-ray diffractograms of the (a) as cast and (b) heat treated alloy NbSiTiHf-5Al and (c) cast and (d) heat treated alloy NbSiTiHf-5Al-5Cr. The non-indexed peaks correspond to un-identified phase(s).

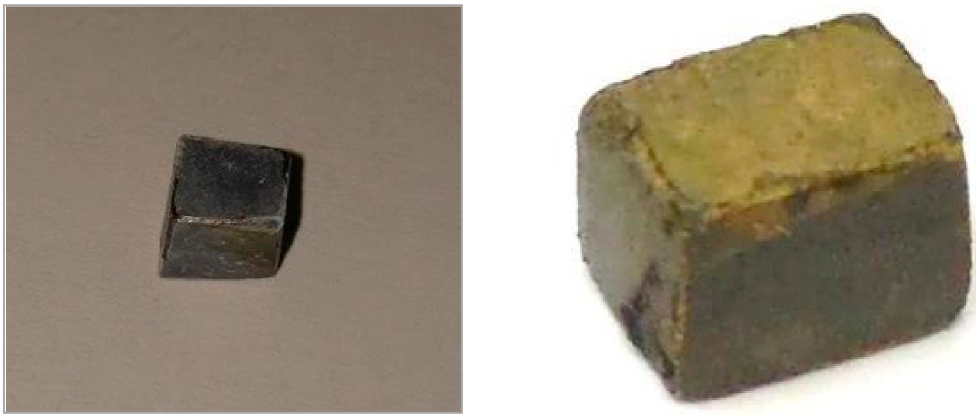

(**a**) (**b**)

Figure S2. Specimens of the alloys NbSiTiHf-5Al (a) and NbSiTiHf-5Al-5Cr (b) after 100 h isothermal oxidation at 800 °C.

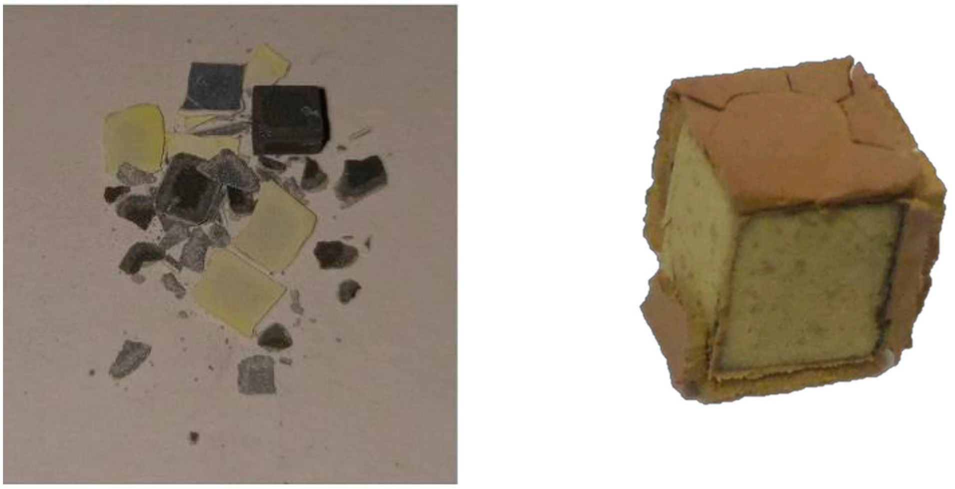

(**a**) (**b**)

Figure S3. Specimens of the alloys NbSiTiHf-5Al (a) and NbSiTiHf-5Al-5Cr (b) after 100 h isothermal oxidation at 1200 °C.

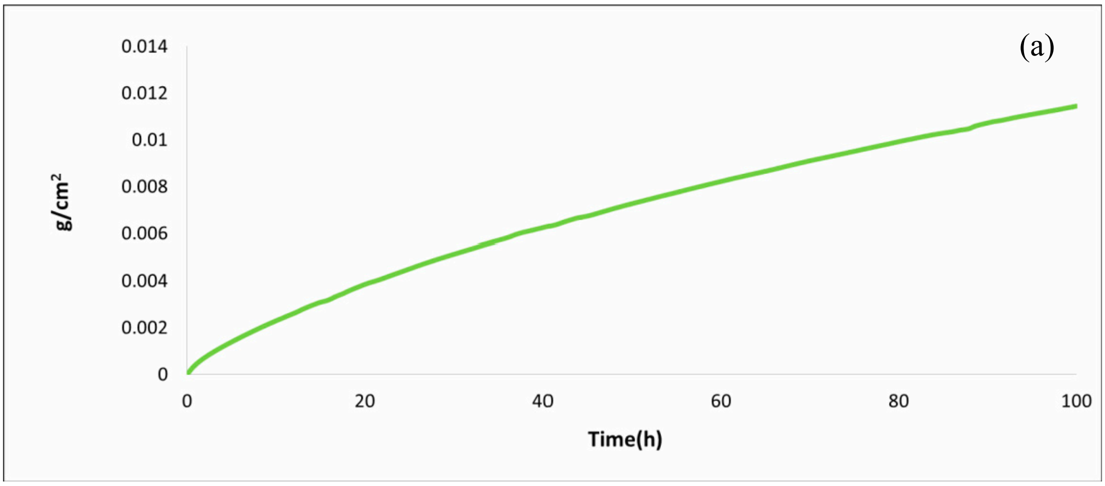

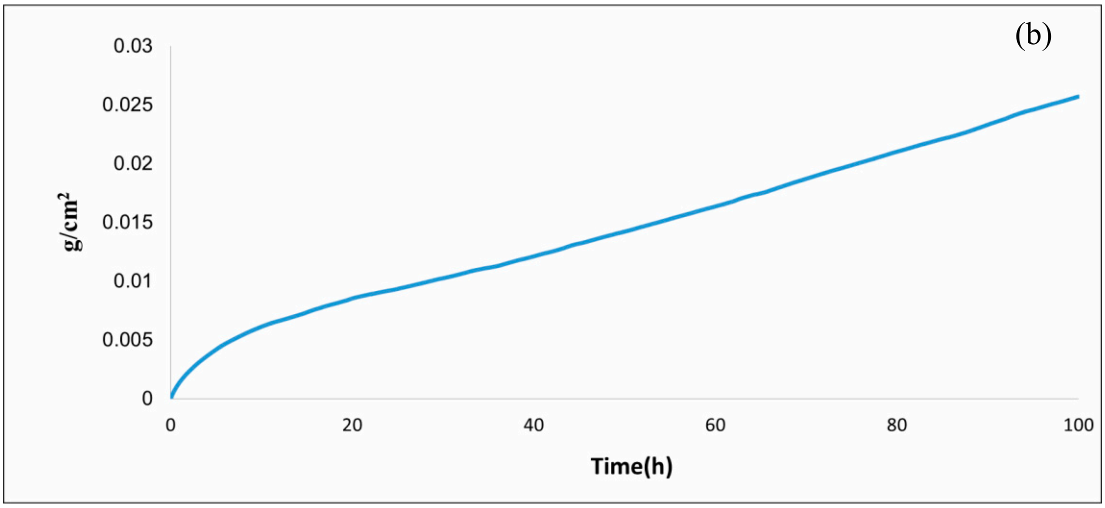

Figure S4. Isothermal oxidation data for the alloy NbSiTiHf-5Al (a) 800 °C, (b) 1200 °C.

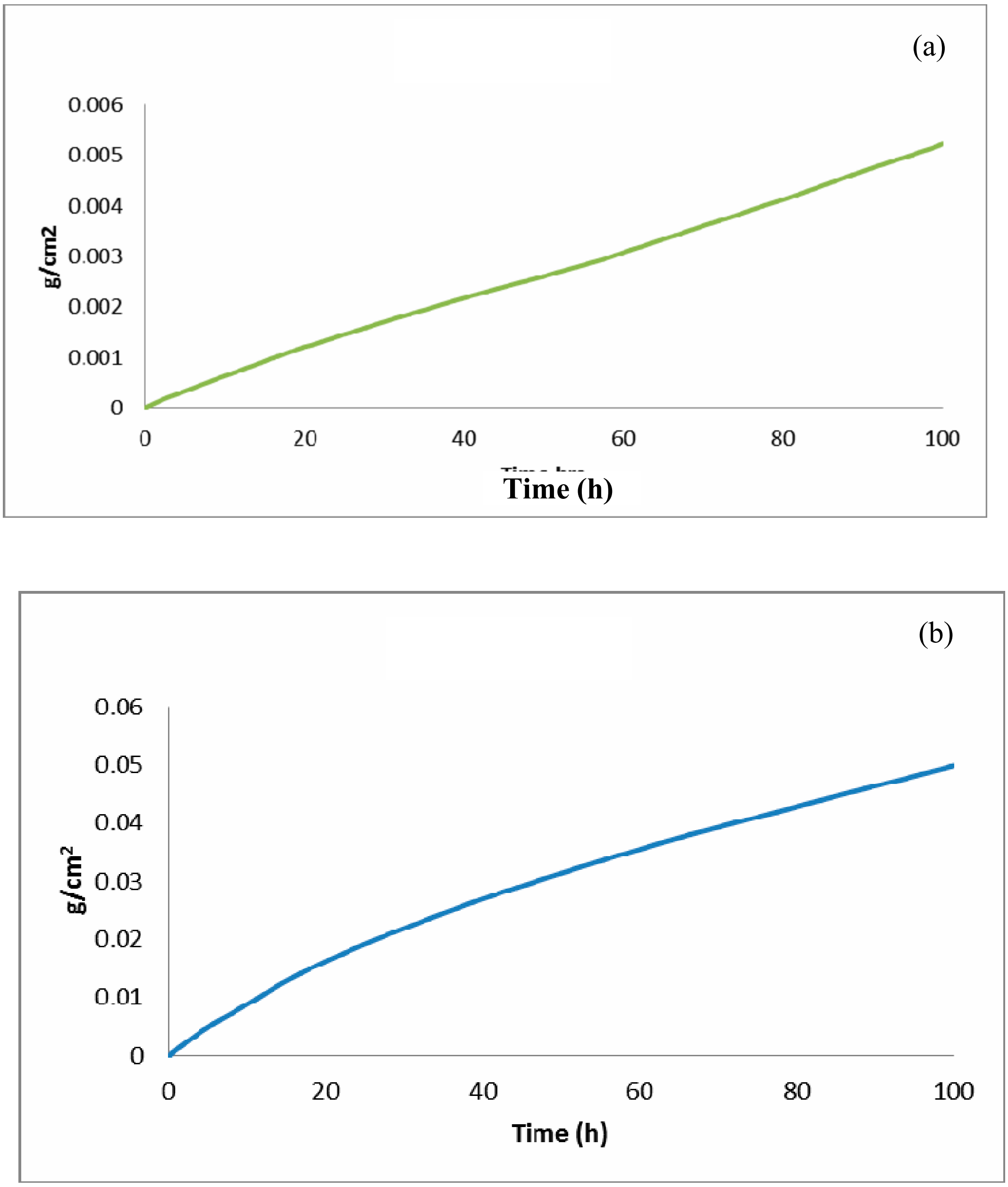

Figure S5. Isothermal oxidation data for the alloy NbSiTiHf-5Al-5Cr (a) 800 °C, (b) 1200 °C.

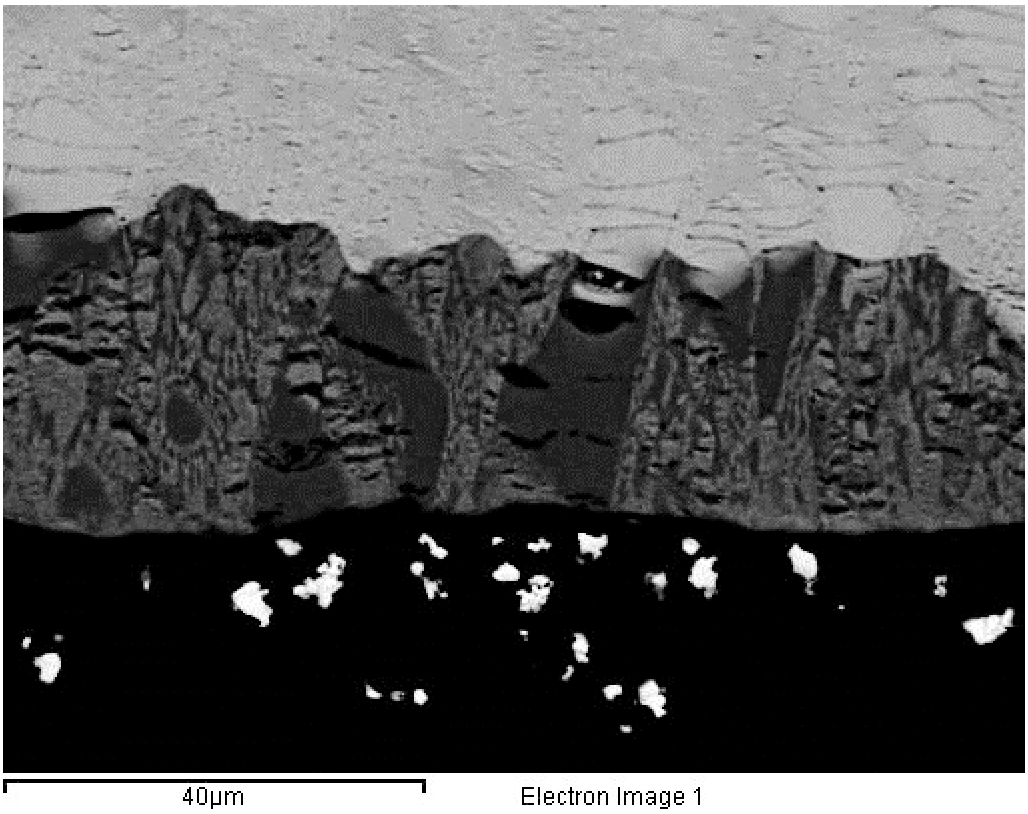

(**a**)

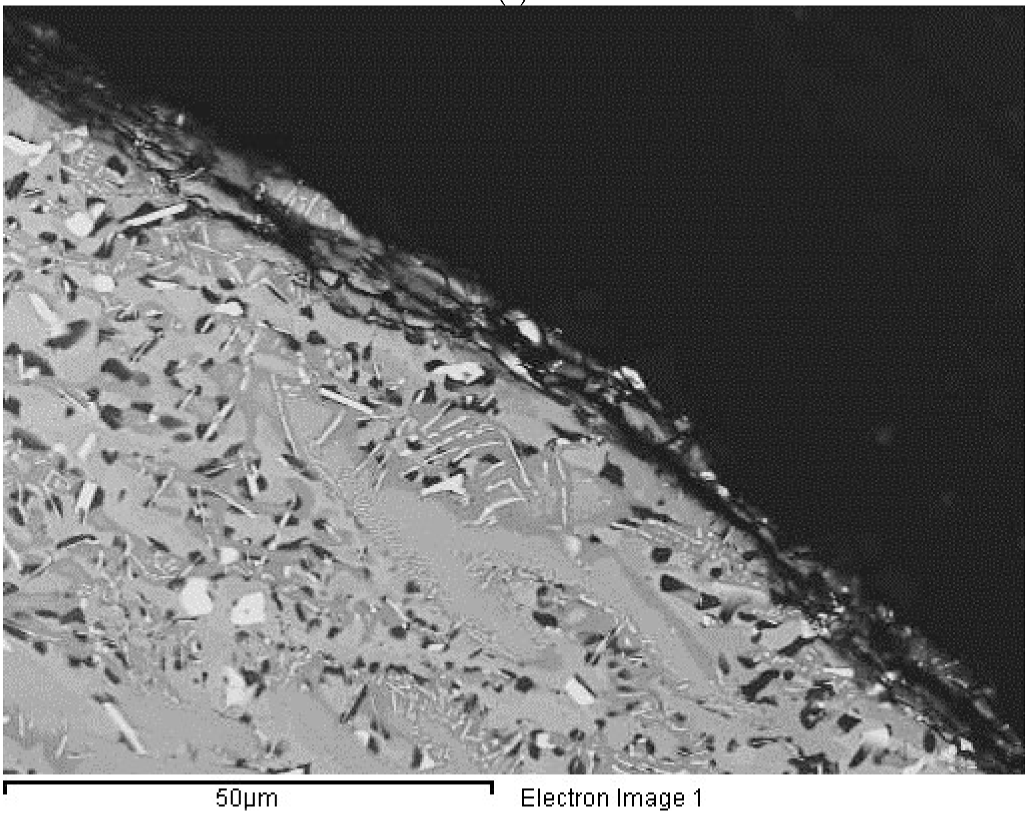

(**b**)

Figure S6. BSE images of cross sections of the alloy NbSiTiHf-5Al after isothermal oxidation (a) at 800 °C and (b) at 1200 °C.

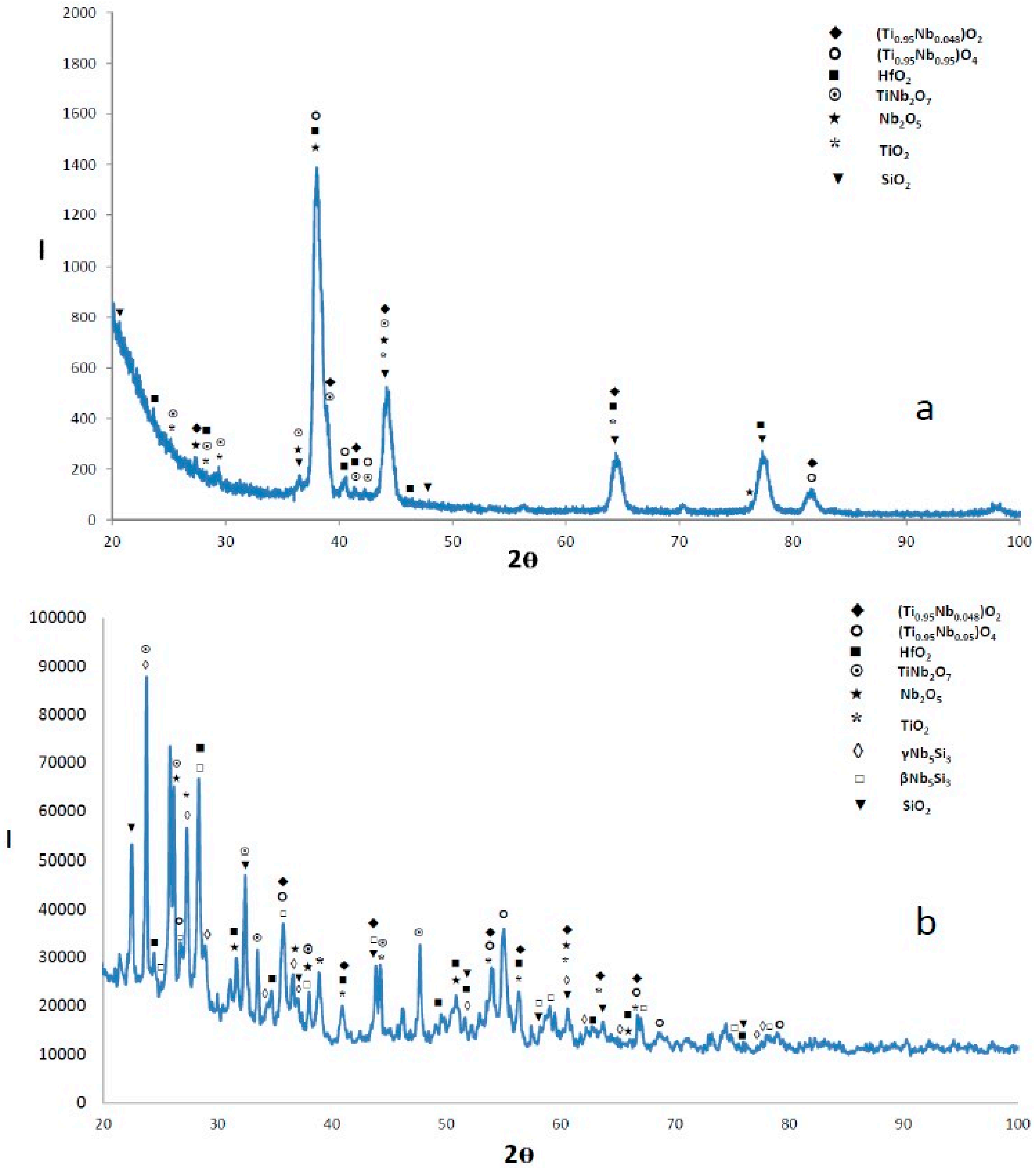

Figure S7. XRD data for the alloy NbSiTiHf-5Al (a) glancing angle data (GXRD, θ=5 degrees) for 800 °C, (b) powder XRD data from the spalled off scale at 1200 °C.

© 2018 by the authors. Submitted for possible open access publication under the terms and conditions of the Creative Commons Attribution (CC BY) license (http://creativecommons.org/licenses/by/4.0/).
